# Coval: Improving Alignment Quality and Variant Calling Accuracy for Next-Generation Sequencing Data

**DOI:** 10.1371/journal.pone.0075402

**Published:** 2013-10-08

**Authors:** Shunichi Kosugi, Satoshi Natsume, Kentaro Yoshida, Daniel MacLean, Liliana Cano, Sophien Kamoun, Ryohei Terauchi

**Affiliations:** 1 Iwate Biotechnology Research Center, Kitakami, Iwate, Japan; 2 Kazusa DNA Research Institute, Kisarazu, Chiba, Japan; 3 The Sainsbury Laboratory, Norwich Research Park, Norwich, United Kingdom; J. Craig Venter Institute, United States of America

## Abstract

Accurate identification of DNA polymorphisms using next-generation sequencing technology is challenging because of a high rate of sequencing error and incorrect mapping of reads to reference genomes. Currently available short read aligners and DNA variant callers suffer from these problems. We developed the Coval software to improve the quality of short read alignments. Coval is designed to minimize the incidence of spurious alignment of short reads, by filtering mismatched reads that remained in alignments after local realignment and error correction of mismatched reads. The error correction is executed based on the base quality and allele frequency at the non-reference positions for an individual or pooled sample. We demonstrated the utility of Coval by applying it to simulated genomes and experimentally obtained short-read data of rice, nematode, and mouse. Moreover, we found an unexpectedly large number of incorrectly mapped reads in ‘targeted’ alignments, where the whole genome sequencing reads had been aligned to a local genomic segment, and showed that Coval effectively eliminated such spurious alignments. We conclude that Coval significantly improves the quality of short-read sequence alignments, thereby increasing the calling accuracy of currently available tools for SNP and indel identification. Coval is available at http://sourceforge.net/projects/coval105/.

## Introduction

Next-generation sequencing (NGS) technology has enabled us to determine whole genome sequences and structures rapidly and inexpensively, including DNA polymorphisms, gene structures, and epigenetic alterations, by producing massive amounts of short reads. The current NGS methodology involves a substantially higher rate of error in reads relative to the Sanger sequencing method. The sequencing error occurs mainly due to technical problems including suboptimal discrimination of fluorescent dyes assigned to different nucleotides and mutations incorporated during the PCR amplification phase of library preparation [1]. The sequencing errors are likely to occur both in a sequence-dependent and a sequence-independent manner. Nakamura et al. have recently reported that GGC-containing genomic regions are prone to sequence-specific errors in Illumina sequencing reactions [2]. This phenomenon may be common in polymerase-catalyzed sequencing systems; a similar bias in sequencing errors has been observed in the 454 GS-FLX system [3]. In addition, the short length (36–110 bp) of the sequence reads often leads to misalignment of the reads to unrelated positions in a reference genome. This is particularly problematic in organisms with genomes containing a large proportion of repetitive sequences. These problems all hinder the accuracy of determination of genomic structures, including DNA polymorphisms, through the alignment of NGS short reads with a reference genome.

A number of short-read alignment tools (aligners), including BWA [4], MAQ [5], SSAHA2 [6], SOAP2 [7], Bowtie [8], NovoAlign (http://www.novocraft.com), RMAP [9], BFAST [10], SHRiMP [11], and Stampy [12] have been developed. Many of these use 20–35 bp substring (seed) sequences within reads for matching with a reference sequence to enable fast and efficient alignment, with algorithms based on hash tables and suffix/prefix tries [13]. The seed-based alignment method increases the probability of finding the best match position and sequence variants in the reference sequence by allowing only a few mismatches between the seed and the reference sequence.

Single nucleotide polymorphisms (SNPs) and insertions and deletions (indels) can be detected from alignment data using a number of freely available SNP/indel calling tools such as MAQ/SAMtools [14], SOAPsnp [15], Crossbow [16], Atlas-SNP2 [17], VarScan [18], [19], Slider II [20], SNPSeeker [21], GATK [22], SeqEM [23], SNVMix [24], Sniper [25], SomaticSniper [26], GeMS [27], GenomeComb [28], DBM [29], Dindel [30], and also using structural variant callers, such as Pindel [31], SvSeq [32], AGE [33], and SV-M [34]. These tools filter out potentially miscalled variants and only call reliable variants. Currently adopted methods of calling SNPs and indels are mainly based on three algorithms; one is to set thresholds empirically for several variables of aligned data such as read depth, base quality, and allele frequency; second relies on a probabilistic measurement of calling accuracy of a variant using a Bayesian approach with statistically measured prior probabilities (e.g., frequencies of SNPs or error occurrence depending on base quality); third is based on a machine leaning method utilizing a support vector machine classifier. For Dindel, GATK, recent versions of SAMtools, and an SRMA realigner [35], calling of small indels has been improved by creating a local realignment around the initially called indel positions. Irrespective of the differences in the calling methods, all of these tools attempt to increase specificity (decrease false positive calls attributable to sequencing and alignment errors) while increasing sensitivity (successful calling of true positives) in the alignment data.

In this study, we show that all of the available short read aligners capable of handling indels (gap-aligners), such as BWA, MAQ, NovoAlign, and BFAST, align a large fraction of the reads with a high number of mismatches. We demonstrate that alignments and DNA variant calling can be significantly improved by correction and filtering of mismatched reads.

## Materials and Methods

### Illumina Short Read Alignment

DNA libraries from rice (*O. sativa* Japonica group cv. Nipponbare) leaves were prepared with the Paired-End DNA sample Prep Kit (Illumina, San Diego, CA, USA), and were sequenced using the Illumina GAIIx platform to produce over 126 million 75 bp paired-end reads with an average insert size of 270±20 bp. Base calling and filtering of low quality bases were performed using sequence control software (SCS) real-time analysis, BCL converter, and the GERALD module (Illumina, San Diego, CA, USA). The rice reads have been deposited in the DDBJ Sequence Read Archive [DDBJ:DRA000470]. The base quality distribution of the reads was examined using PRINSEQ [36]. Seventy-two million 100 bp Illumina GAII paired-end reads of *C. elagans* WS220 [Run SRR065388 from Study SRP003487] and 745 million 76 bp Illumina GAII paired-end reads (in part, containing 54 bp and 104 bp paired-end reads) of mouse 129S5/SvEvBrd [Run ERR007818∼ERR007841 from Study ERP000036] were downloaded from the NCBI read sequence archive (http://www.ncbi.nlm.nih.gov/Traces/sra/). For experiments with trimmed reads, we treated 63 million 75 bp paired-end rice reads using Condetri with options ‘–minlen = 35–ml = 5–lq = 5 or = 10′ and Trimmomatic with options ‘ILLUMINACLIP:GAII:adapter.fa: 1∶40∶15 LEADING:3 TRAILING:3 MINLEN:50 SLIDINGWINDOW:2∶10, :2∶15 or :2∶20′ to produce reads from which the 3′ low quality regions were trimmed. The reference genomes used for alignment were as follows: IRGSP build 5 of rice (*O. sativa* cv. Nipponbare), mouse 129S5/SvEvBrd consensus genome sequence, and *C. elegans* WS220 genome, which were downloaded from RAP_BD (http://rapdb.dna.affrc.go.jp/), the mouse genomes project site (http://www.sanger.ac.uk/resources/mouse/genomes/) of the Wellcome Trust Sanger Institute, and NCBI (ftp://ftp.ncbi.nlm.nih.gov/genomes/), respectively. Assemblies of the mouse consensus sequence were combined to make one contig for each chromosome. The short reads and reference genomes used in this study are listed in Table S1. We used BWA v0.5.9 by default paired-end or single-end mode for alignment unless otherwise stated. For paired-end alignment with SOAP2 v2.21, Bowtie 2 v2.0.0-beta7, and Stampy v1.0.15, with default parameters, were used, and the options ‘–a 400’, ‘–r A 2′, and ‘–rtype solexa –best 1′ were used for alignment with MAQ v0.1.7, NovoAlign v2.07.18, and SSAHA2 v2.5.4, respectively.

### Development of Coval Tools

Coval is implemented as a set of Perl programs. An outline of the Coval pipeline and a schematic description of the Coval-Refine algorithm are illustrated in Figure S1.

#### SAM/BAM alignment-refining tool (Coval-Refine)

The Coval-Refine tool removes reads with mismatches that exceed the number specified by users and whose base-call qualities are lower than the value specified by users, after the local realignment procedure that repairs mismatched alignments in the ‘basic mode’. Coval-Refine corrects potential sequencing errors in reads after local realignment, and then removes reads with mismatches that exceed the number specified by users in the ‘error correction mode’. In this mode, mismatch reads with a larger number of mismatches than 10% of read length are removed before realignment. The realigned reads are error-corrected, based on base quality and/or allele frequency of mismatch bases. The quality-based correction is a strategy to correct mismatch bases when the mean quality of non-reference bases supported at the site is lower than a minimum quality value (10 by default) specified by users, and the frequency-based correction is to correct mismatch bases when the non-reference allele frequency is lower than a minimum allele frequency (0 by default) specified by users (see Figure S2 for the error-correction algorithm in more detail). If there are multiple non-reference alleles in a mismatch site, mismatch bases with lower allele frequencies at the site are corrected to match the corresponding reference base. For data from pooled samples, allele frequencies are calculated for each sample and bases with the non-reference allele frequency lower than 0.8 for homozygous samples or lower than 0.3 for heterozygous samples are corrected to reference bases by setting ‘msamp’ option. By default in the error correction mode, when the first mate is filtered out the second paired-end mate is also removed (which is also selectable with option ‘fpair’). In both the modes, the filtering also removes discordant paired-end reads, either with mates that are not aligned or with a distance between the mates that exceeds the mean distance plus five standard deviations, which is automatically calculated. In our simulation tests, however, the removal of discordant paired-end reads only slightly improved the accuracy of SNP and indel calling. Removal of the discordant paired-end reads and selection of a threshold filtering length of the read distance are selectable as options, which may be useful for a downstream analysis such as structural variant finding. Coval-Refine also removes reads containing more than two indels or containing soft-clipped ends at both termini. The realignment function realigns mismatch reads overlapping with an indel, which generally contain multiple mismatches in their terminal regions due to the failure of the gapped alignment to support the indel. Coval-Refine also removes reads containing any mismatch within their terminal 2 bp regions only when the local realignment is disabled, which is selectable as an option.

The filtering tool finds mismatches in reads and counts the number of mismatches by comparing the read sequence with the corresponding region of the reference genome. It does not use the MD tag for mismatch information in SAM alignment data for initial seeking of mismatch, enabling application to other kinds of aligners. In the filtering of mismatch reads, the presence of an indel or a soft-clipped end is regarded as one mismatch; for example, a specified two mismatch threshold goes down to a one mismatch threshold for reads containing a single indel. The maximum number of allowable mismatches and maximum rate of mismatches in a read are controlled by the ‘num’ (default is 2) and ‘mrate’ options, respectively. The maximum number of total mismatches contained in two paired reads can be also specified by the option ‘fnum’ (default is 1.7-fold of the number specified with ‘num’). The balance between TPR and FPR can be also controlled by the mismatch-counting with the base quality of mismatched bases; counting as ‘mismatch’ only mismatched bases with base call quality less than the value specified with the option ‘minq’.

Short-read alignment tools often misalign reads around indels, which in many cases results in mismatches in the terminal regions of the reads, local realignment around indels improves the accuracy of indel calling, as previously reported [22], [30], [35]. The local realignment function of Coval first stores the indel information and mismatch-containing reads in the alignment data. Second, it realigns mismatched reads such that the extracted indel positions overlap with the target indel region. The realignment is conducted by shifting the 5′ or 3′ portion of a read at the target indel site by the length of the indel size. When the total mismatch number of a read is decreased or unchanged by the realignment procedure, the realigned state is accepted and stored in an alignment result. The reads to be realigned are restricted to reads with at most one indel or one soft-clipped end, because of the complexity of the realignment process. For reads without indels or soft-clipped ends, only mismatch-containing reads are realigned because the realignment of non-mismatch reads worsens the variant calling accuracy.

The treatment of an alignment file containing 50 million reads with Coval-Refine should be accomplished within a few hours but it will take twice or more time when setting –mfreq or –msamp in the error correction mode.

#### Variant calling tool (Coval-Call)

The variant calling tool is a filtering tool to call SNPs and indels from a pileup or SAM file. This filtering is based on the following options for empirical settings: (1) ‘num’, the number of reads with a non-reference base at the called site (default is 2), (2) “freq”, the frequency of reads supporting a called allele in the total number of reads covering the site (default is 0.8), (3) ‘qual_ave’, the average base quality of non-reference bases at the called site (this is only for SNPs, default is 20), ‘qual_base’, the minimum base quality of a non-reference base (default is 3) (4) ‘maxr’, the maximum read number covering non-reference alleles. For heterozygous SNP calling, for example, when ‘freq’ is specified to 0.2, a called allele with ‘freq’ <0.9 is controlled by another option, ‘tnum’, the minimum number of reads supporting a heterozygous non-reference allele (default is 3), whereas an allele with ‘freq’ ≥0.9 is controlled by ‘num’. For SNP calling, SNPs that are present within 3 bp upstream or 3 bp downstream of a homozygous indel are not called. For ‘freq’ calculation for indel calling, the number of reads covering the site that is represented at the eighth column of a pileup file has been subtracted from the number of reads whose 3′ termini (including soft-clipped ends) are located at the site. DNA variant calling from a pileup file containing 1.5 million variants would take about 1 min.

#### Genome-simulator (Coval-Simulate)

Genome-simulator was designed to incorporate SNPs and 1 to 6 bp indels randomly into a reference genome, evenly distributing the number of SNPs and indels specified by users, according to the length of each chromosome. Moreover, SNP bases to be substituted are set to reflect the base frequency of naturally occurring SNPs; transitions are 4-fold more frequent than transversions, as previously observed [37]. Indel lengths to be inserted are also set to reflect naturally occurring frequencies. We set the frequency of indels with different lengths as follows: 66% were 1 bp indels, 17% were 2 bp indels, 7% were 3 bp indels, 7% were 4 bp indels, 2% were 5 bp indels, and 1% were 6 bp indels. These percentages were based on observed frequencies in the human genome [38]. The frequencies of both the SNPs and indels were roughly identical to those from our resequencing results for different rice cultivars.

### Simulation Tests

A simulated rice genome containing 816,408 artificial SNPs (corresponding to 0.2% of the rice genome) and 76,100 1 to 6 bp indels (corresponding to 0.02% of the rice genome) was created with the Coval-Simulate tool. This simulated genome was then aligned with the experimentally obtained rice reads using BWA or other aligners. The published rice genome sequence was slightly different from our rice cultivar, even though it was the same Nipponbare strain, and this would be expected to cause a high background of erroneous SNP/indel calling in the simulation test. Thus, we needed to substitute or modify the rice reference bases with those of reliable SNPs and indels that were called between the reference and the reads from our rice strain. This reference modification step involves the incorporation of false SNPs/indels that could affect a subsequent simulation analysis. Our simulation analysis, by different strategies, for reference modification suggested that we can minimize the incorporation of false positives when using an aligner that is different from that used in the simulation tests, and that we can select SNPs/indels in a strict filtering condition (Table S2). We aligned Nipponbare reads using NovoAlign (output option: –r A 1), and called 3,411 homozygous SNPs and 2,279 homozygous small indels with the Coval-Call tool. The settings were ‘coval call –num 3–freq 0.9–qual 20′, where the minimum number of reads supporting the non-reference allele was 3 and the minimum frequency of the non-reference allele was 0.9. The rice reference genome was modified by substitution with these SNPs and by insertion/deletion of the regions corresponding to the indels. For the simulation test with mouse data, we used 745 million mouse paired-end reads obtained from the Sequence Read Archive (http://www.ncbi.nlm.nih.gov/Traces/sra/) and the consensus assembly of the mouse strain 129S5/SvEvBrd as reference, and created a simulated mouse genome containing artificial SNPs and indels (0.2% and 0.02% of the genome, respectively) using a modified reference, wherein the bases of the original reference had been replaced with reliable endogenous SNPs and indels detected between the reference and the reads. The simulated genome was aligned with the mouse reads using BWA, yielding coverage with an average read depth of 21×, and DNA variants were called with or without the Coval-Refine tool. In a similar way, 1,186 homozygous SNPs and 1,350 homozygous indels of *C. elegans* were called under a stringent filtering condition using about half (34 million) of the downloaded reads, and were substituted for the bases of the reference genome. Using these modified genomes and the Coval simulation tool, we created simulated genomes containing artificial SNPs and 1 to 6 bp indels corresponding to 0.2 and 0.02% of each genome, respectively.

For a simulation test for heterozygous SNP calling, we created 120 million 75 bp paired-end simulated reads using a dwgsim simulator (http://sourceforge.net/apps/mediawiki/dnaa/index.php?title=Whole_Genome_Simulation), which contained 447,626 heterozygous and 222,167 homozygous SNPs, and 90,041 homozygous 1 to 10 bp indels, together with artificial errors showing an increasing error rate toward the 3′ termini of reads (0.12% to 0.8%). Simulated reads were also created using pIRS and MAQ (‘maq simutrain’) simulators, where the read quality, error distributions, and GC content-coverage bias (for pIRS) were reflected by calibrated data for the rice reads.

Filtering of alignments with Coval-Refine was carried out with the default options (e.g., maximum number of allowable mismatches in a read and two paired reads and is two and three, respectively) unless otherwise stated. No variant was called at positions of ambiguous reference bases.

### SNP and Indel Calling

To extract DNA variants from alignment data, we used SAMtools. PCR-duplicated reads were removed from BAM alignment files with the command ‘samtools rmdup’, and pileup files containing only variants were produced with the command ‘samtools pileup –vcf’. Alternatively, DNA variants were called directly from SAM files with the ‘coval call-sam’ command of the Coval tool. To call homozygous SNPs or small indels, the pileup files were filtered with the Coval command ‘coval call’ with the default options (–num 2–freq 0.8–qual 20) and by specifying 3-fold of an average read depth for the option –maxr (Table S3).

Detailed parameters used with the SNP or indel callers are shown in Table S3. Homozygous SNP calling with the available variant callers in the rice simulation study was conducted as follows: Samtools.pl varFilter (options: –d 2–D 35–S 10) was piped with awk ‘$4∼/[ACGT]/&&$3! = “*”&&$6> = 20′. SAMtools mpileup (options: –uBf for SNP calling and –uf for indel calling, -Q (default: 13)) was piped with bcftools view (options: –vc –i 0.1–t 0.002) to produce variant call format (VCF) files. VCF files were further filtered to select variants that had at least two reads supporting the variants and that showed a minimum allele frequency of 0.8 and a maximum read depth of 35 at called sites. Results from GeMS were further filtered by removing SNPs with ‘largest posterior probability <0.8′. For the GATK pipeline (v2.2), ‘base quality score recalibration’, ‘local realignment’, and ‘UnifiedGenotyper’ were successively applied to BAM alignment files that had been pre-filtered with a ‘bwa rmdup’ command. The base quality score recalibration of GATK was conducted using all of the SNP sites that were introduced into the simulated rice genome as known sites. We did not use ‘variant quality score recalibration’ of GATK because, when using high quality SNPs/indels that were called with Coval-Refine and Coval-Call [–num 6–freq 0.9–qual 30] (20% of the called variants) as both true and training sets and unfiltered SNPs/indels (50% of totally called variants) as another training set, the result was worse than when a simple filtering method was used. This was likely due to incompatibility of the recalibration modeling with our simulation system. Thus, the UnifiedGenotyper-generated VCF files were filtered by selecting variants supported by at least two reads and that showed a minimum allele frequency of 0.8.

## Results

### Reads with a Large Number of Mismatches Found in Short Read Alignments

We show that alignment data from short reads contain many reads with a large number of mismatches, even when the maximum number of mismatches between the read seed and reference sequence has been set to 2. Figure S3 shows examples of such alignment data with *Oryza sativa*, *Arabidopsis thaliana*, *Caenorhabditis elegans*, and *Mus musculus* illumina reads. About 3% of the aligned reads had more than two mismatches (Table 1), and over 72% of the mismatches were located in the 3′-terminal regions of the reads, excluding the 35 bp seed. Many of the mismatches correlated with low-quality base calls, which tend to be pronounced in the 3′ regions, although the quality of the base calling in the rice reads was high, with a median quality score of 36 at the 3′-termini (Figure S4). Alignment results obtained with gap-aligners, including BWA, MAQ, NovoAlign, and BFAST, contained high-mismatch reads, whereas those with gap-free aligners, such as SOAP2 and Bowtie (but not its latest version, Bowtie 2 [39]), did not show such patterns, even in the “seeding” mode (Table S4). By contrast, the alignment data from artificially generated short reads that were made using a pIRS [40] or dwgsim simulator (http://sourceforge.net/apps/mediawiki/dnaa/index.php?title=Whole_Genome_Simulation) did not contain such high-mismatch reads (Figure S3E), indicating that simulated reads do not produce the erroneous alignment observed in the experimentally obtained reads.

**Table 1:** Number of mismatches in aligned reads.

Mismatch number	Percentage of mismatched reads in aligned reads (%)^a^				
	rice	Arabidopsis	nematode	mouse	simu-dwgsim
1	8.6	9.1	19.0	15.7	24.4
2	2.7	2.1	4.5	5.9	4.1
3	1.6	1.4	1.7	3.6	0.45
4	1.2	0.9	1.0	2.7	0.037
5	0.4	0.2	0.6	1.4	0.001
>5	0.8	0.4	0.1	2.8	0.0001
Error rate	0.42	0.33	0.41	1.1	0.45

a The percentage of reads with mismatches, out of the total number of aligned reads for each species or simulated reads. Aligned reads are paired-end reads of 100 bp for nematode, 76 bp for mouse, and 75 bp for the others. Artificial reads reflecting the error tendency of the rice reads were generated with a dwgsim. The total error rates (%) are indicated in the last line.

We also noticed that many of the high-mismatch reads were clustered especially within and/or around low read-coverage regions (Figure S3). When we counted the average number of mismatches per read in the rice read alignment, which had an average read depth of 11.5×, we found that low coverage regions with read depth ≤3× contained an 8-fold larger number of reads carrying more than two mismatches compared with those with read depth >6× (Figure S5). Similar observations were made in the case of alignment of paired-end reads in *Arabidopsis* and nematode alignments but not in alignments with mouse and simulated reads. Overall, these observations suggest that low read-coverage regions tend to have a higher incidence of misalignment and miscalling of DNA variants. Indeed, we confirmed in a simulation test (see below) using rice and nematode reads that the regions covered by lower numbers of reads exhibited significantly lower accuracy in calling SNPs (Table S5).

### Simulation System (Coval-Simulate and Coval-Call) to Evaluate SNP/indel Calling Accuracy Using Experimentally Determined Data

For reliable calling of SNPs/indels, a simulation system is required that faithfully reflects observed, real read-error properties to evaluate the accuracy of SNP/indel calling. We conclude from the error model in currently available read simulators that they do not reproduce the complex patterns of error seen in real read sets (Table 1; Figures S3 and S5). For this purpose, we first developed a tool for simulated genome alignment using real read data and a tool for DNA variant calling, as a component of the alignment-improving and SNP/indel calling pipeline Coval. The Coval simulation tool (Coval-Simulate) creates a reference genome containing computationally introduced SNPs at a defined rate and 1 to 6 bp indels. SNP base-changes and indel length were set to follow empirical frequency distributions (see Materials and Methods). The Coval SNP/indel calling tool (Coval-Call) filters and calls SNPs and small indels by checking for three main factors: read depth, base quality, and allele frequency at the called allele sites, as described [41] (see also Materials and Methods). As a first trial, we created a simulated rice genome, where SNPs (7.4 × 10^5^, which corresponds to 0.2% of the genome) and 1 to 6 bp indels (7.4 × 10^4^) were artificially introduced into a modified Nipponbare rice reference genome using Coval-Simulate (see Materials and Methods). To create the alignment, we used experimentally obtained sequence reads from Nipponbare (6.3 × 10^7^ 75 bp paired-end reads from the rice genome). To evaluate the SNP calling accuracy, candidate SNPs/indels were extracted from the alignment data using SAMtools (‘samtools pileup’ command) and then called using the Coval-Call tool. Since Coval-Simulate allowed us to know the exact position of artificially introduced SNPs/indels, we could evaluate how many were recovered by alignment of short reads followed by calling with various software including Coval-Call.

To confirm the usefulness of our simulation system, we compared the SNP/indel calling accuracy in our system with that in a conventional simulation test using artificial reads created by short-read simulators. A total of 6×10^7^ simulated 75 bp paired-end reads generated with dwgsim and pIRS were aligned to the wild-type rice reference (see Materials and Methods for detail). SNPs/indels were then called using Coval-Call with the same filtering thresholds as in our simulation method. The results from simulated reads showed much higher accuracy for calling SNPs and indels than those from real reads (Table S6). These results suggest that the simulation system using artificial reads tends to give a level of accuracy of SNP calling that is too high and that does not reflect the real experimental situation. Thus, our system based on real experimental condition is more suitable for evaluating the SNP/indels calling performance of available tools.

### Development of Coval-Refine, a tool that Improves Alignments by Removing and Correcting Multiple-mismatch Reads

To improve alignments with the high-mismatch reads, we first tried to trim the 3′ portions of reads that harbored consecutive low-quality bases. When we trimmed the 3′ low-quality regions of the rice reads, with Illumina Phred-like quality of <5, using the Condetri [42] and Trimmomatic (http://www.usadellab.org/cms/index.php?page=trimmomatic) read-trimming tools, the alignment with the 3′-trimmed reads, however, did not significantly lower the false positive rate (FPR), and instead lowered the true positive rate (TPR) (Table S7). These results are consistent with previous observations by Liu et al. [43], indicating that the observed mismatches may not be solely attributable to low-quality sequencing results. We developed an alignment-refinement tool, Coval-Refine, by filtering, correcting, and local realigning mismatched reads (see Materials and Methods and Figure S1 for details). To minimize the loss of true positives through filtering, Coval-Refine was designed to correct sequencing errors that were judged from their base call qualities and the allele frequency at the mismatch sites (‘error correction mode’). The Coval-Refine treatment in the error correction mode increased the TPR by ∼1.5% compared with that in the basic mode without error-correction with only a small increase in FPR for both SNP and indel calling (Figure S6). The application of Coval-Refine in the error correction mode to alignment data with various gap-aligners decreased the FPR by 60–77% for SNP calling and by ∼60% for indel calling, whereas decreasing the TPR by only 1–2.5% for SNP calling and increasing the TPR by ∼40% for indel calling (Figure S7). The increase in TPR for indel calling is mainly due to the effect of the local realignment function of Coval-Refine. It is known that misaligned indels cause miscalling of SNPs around the indels, but the FPRs in SNP calling were almost unchanged by the local realignment probably due to the removal of misaligned reads, the basic function of Coval-Refine. This tool could be applied robustly to alignment data consisting of different numbers of reads (Figure S8) and also to simulated genomes containing a wide range of SNP (Table S8) and indel (Table S9) content by adjusting the filtering threshold number of mismatch contained in reads.

Some fractions of real DNA variants would not be evenly distributed across the genome but are clustered. The clustered variants could be missed when several filtering thresholds of mismatches are specified for Coval-Refine. Out of the variants introduced into the simulated rice genome in this simulation test, 3.1% are present in clustered regions where at least three variants reside within a range of read length (*i.e*., 75 bp). The missed variants (false negatives) could be minimized by an increase of the filtering threshold of mismatch (i.e., –fnum 4)(Table S10).

### Coval-Refine Improves the Accuracy of Heterozygous SNP Calling in a Single and Pooled Sample

Coval-Refine was also effective for heterozygous SNP calling in a simulation test using simulated rice reads containing heterozygous SNPs generated by the dwgsim and pIRS tools (see Materials and Methods and Table S11). However, the simulation test with the artificially generated reads resulted in too high TPR and too low FPR, as also observed for homozygous SNP calling. To reflect the property of real data more faithfully, we mixed the experimentally obtained rice reads with artificially simulated reads that were generated using the simulated rice reference in a different ratio and aligned to the simulated rice reference. The ratios of the rice real reads to be mixed were 12.5%, 25%, and 50%, reflecting the frequency of heterozygous alleles. As expected, SNP calling from these alignments resulted in a significantly higher number of false positives than those only with artificially simulated reads (Table S12). These false positives were effectively reduced by the treatment of the alignments with Coval-Refine.

Coval-Refine was designed to treat the aligned reads in pooled samples. The read mixtures used for the above analysis was used as pooled samples, where the experimentally obtained read set (sample) was discriminated from the other read set using the RG tag in the alignment. The alignment refinement for each sample in the pooled alignment data by Coval-Refine enabled more accurate SNP calling than those of heterozygous SNP calling from the single sample data (Table 2 and Table S12), indicating the utility of Coval-Refine on pooled samples.

**Table 2:** Calling accuracy of SNPs from alignment data containing multiple samples.

Sample (%)^a^	Homo/Hetero^b^	Coval-Refine	SNP calling accuracy	
			True positive rate	True positive rate
50	Homo	−	617,351 (83.4%)	37,535 (5.73%)
		+	605,175 (81.8%)	1,255 (0.21%)
	Hetero	–	542,474 (73.3%)	21,588 (3.83%)
		+	492,574 (66.5%)	5,575 (1.12%)
25	Homo	–	542,399 (73.3%)	21,198 (3.76%)
		+	527,156 (71.2%)	1,619 (0.31%)
	Hetero	–	376,688 (50.9%)	7,345 (1.91%)
		+	348,833 (47.1%)	2,849 (0.81%)
12.5	Homo	–	375,386 (50.7%)	7,242 (1.89%)
		+	361,669 (48.9%)	1,369 (0.38%)

The experimentally obtained rice reads (60, 30, and 15 millions) were mixed with the simulated 75 bp paired-end reads (60, 90, and 105 millions) generated by dwgsim with the rice simulated genome as template, respectively, yielding 120 millions of reads. The read mixtures were aligned to the rice simulated genome, resulting in alignments with average read depth of 24×, and each read set (sample) in the read mixtures was discriminated from the other read set using the RG tag. The SNPs were called using Coval-Call with a maximum of 80 reads covering the called positions, a minimum allele frequency at the called position of 0.2 (for 50% homozygous sample), 0.1 (for 50% heterozygous and 25% homozygous samples), or 0.05 (for 25% heterozygous and 12.5% homozygous samples), a minimum of three reads (for 50% homozygous sample) or two reads (for the others) supporting the called allele.

a Percentage of the experimentally obtained rice read sample in the read mixture.

b Heterozygosity of the experimentally obtained rice read sample (Homo: 0% heterozygosity, Hetero: 50% heterozygosity).

### Coval-Refine Improves the Accuracy of Available Variant Callers

**Figure 1:**
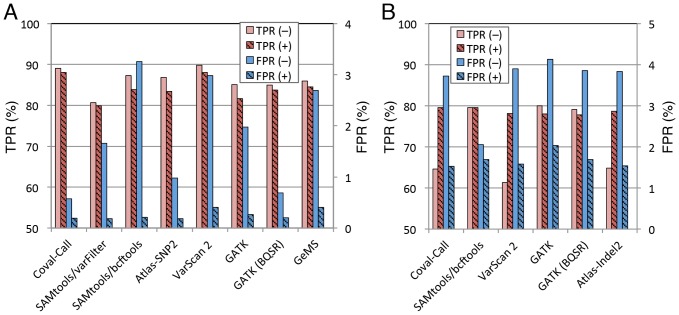
Improvement of SNP/indel calling accuracies of various DNA variant callers by Coval-Refine. (A) SNP calling accuracy with or without Coval-Refine. (B) Indel calling accuracy with or without Coval-Refine. The simulated rice genome was aligned with reads of the real rice genome (experimental reads) using BWA. Alignment data were filtered (+, red striped and blue striped bars) or not filtered (–, light red and light blue bars) with the Coval-Refine component (Coval-Refine, error correction mode), and homozygous SNPs and indels were called using the indicated variant callers. The SNPs and indels extracted by all the callers were further filtered under the same conditions, as described in the text. True positive rate (TPR, the number of successfully called SNPs or indels divided with the number of SNPs or indels introduced into the simulated genome, followed by multiplying with 100) is shown with light red and red striped bars, and false positive rate (FPR, the number of wrongly called SNPs or indels divided with the number of the totally called SNPs or indels, followed by multiplying with 100) with light blue and blue striped bars. The GATK pileline was carried out with (GATK BQSR) or without (GATK) the base quality score recalibration. A variant quality score recalibration in the GATK pipeline was omitted because of its unsuitability for our data. Instead it was replaced by simple filtering: a minimum allele frequency of 0.8 and a minimum allelic read depth of 2 (see Materials and Methods for details).

To compare the accuracy of SNP/indel calling between Coval and other available tools (SAMtools pileup/varFilter, SAMtools mpileup/bcftools, Atlas-SNP2, Atlas-Indel2, VarScan 2, GeMS, and GATK), we used data from the alignment of experimentally obtained rice reads (63 million Illumina paired-end reads) with the simulated rice genome containing artificial mutations. We did not test other SNP/indel callers described in the Introduction section since their specific usage (*e.g*., heterozygous SNP calling from pooled sequencing data) does not fit our simulation experiment. To evaluate the performance of the tools fairly as possible, the SNPs and indels extracted by all the callers were further filtered under the same conditions, with a minimum allele frequency at the called position of 0.8, a minimum of two reads supporting the called allele, and/or a maximum of 35 reads covering the called positions. SNP/indel calling with the Coval-Refine and Coval-Call tools resulted in a TPR of 87.6% and an FPR of 0.18% for SNP calling, and a TPR of 79.6% and an FPR of 1.56% for indel calling (Figure 1; Tables S13 and S14). This SNP calling accuracy, especially the specificity, was higher than that of any of the other SNP calling tools tested (Figure 1A; Table S13). The indel calling accuracy of our tool was also better than any of the other indel calling tools (Figure 1B; Table S14). It is, however, difficult to conclude that our Coval pipeline has the best performance. Because a variant calling accuracy varies depending on sequencing depth [43] and the probabilistic variant-filtering strategies adopted in other tools are optimized for specific species and samples (*i.e.*, human tumors), other tools may not perform well under our simulation conditions. In this analysis, the filtering threshold was set to require at least two reads with non-reference bases at the called variant site. For indel calling the indels supported only by a single read had a significantly low level of FPR in alignment data treated with Coval-Refine (Figure S9). These results indicate an ability of Coval to call DNA variants from low coverage regions accurately.

We then examined whether the enhanced quality of alignment data with Coval-Refine affects the variant calling accuracy of the previously reported SNP/indel calling tools. Application of the Coval-Refine component to other SNP callers considerably lowered the FPRs for other tools by 85–92% without significantly decreasing their TPRs (Figure 1A; Table S13). The FPRs of all the indel callers tested were decreased by 60–82% in the alignment treated with Coval-Refine, and the TPRs of the non-realignment indel callers, Atlas-Indel2 and VarScan 2, was significantly increased by 22 and 28%, respectively (Figure 1B; Table S14). Their overall accuracies in the error correction mode of Coval-Refine were higher than those in the basic mode, while those for mouse data were higher in the basic mode, as shown in the next section. These results indicated that the Coval-Refine tool can improve the SNP and indel calling accuracy of many available DNA variant callers.

### Coval Pipeline Applied to Mouse Alignment Data

**Figure 2:**
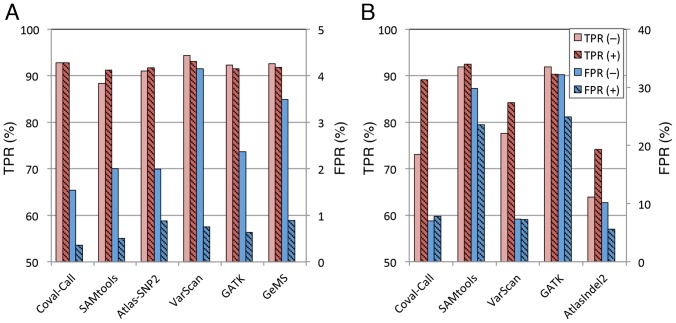
Improvement by Coval-Refine of SNP/indel calling accuracy of variant calling tools for mouse alignment data. (A) SNP calling accuracy with or without Coval-Refine. (B) Indel calling accuracy with or without Coval-Refine. A simulated mouse genome was aligned with real mouse read data using BWA. The alignments were filtered (+, striped bars) or not filtered (–, plain bars) with Coval-Refine. Homozygous SNPs and indels were called with the indicated variant callers under the same conditions as in Figure 1.

To confirm whether the improved variant calling accuracy by Coval is also observed in reads from other organisms, we conducted simulation tests with the mouse genome. We used 745 million mouse paired-end reads obtained from the NCBI Sequence Read Archive (http://www.ncbi.nlm.nih.gov/Traces/sra/) and the consensus assembly of the mouse strain 129S5/SvEvBrd as reference, and created a simulated mouse genome containing artificial SNPs and indels (0.2% and 0.02% of the genome, respectively), as described in Materials and Methods. The simulated genome was aligned with the mouse reads using BWA, yielding coverage with an average read depth of 21×, and DNA variants were called with or without the Coval-Refine tool. The Coval-Refine tool significantly improved the accuracy of SNP and indel calling for the mouse alignment data, compared with calling without Coval-Refine (Figure 2; Table S15). The Coval-Refine treatment decreased the FPR of the called SNPs from 1.54% to 0.36%, while keeping the TPR unchanged. A high level of indel FPRs suggests the presence of many endogenous indels remained to be removed from the reference. A similar improvement of SNP/indel calling accuracy was also observed in a simulation study with the nematode genome and real reads (Table S16). Moreover, Coval-Refine improved the performance of all of the other tested SNP/indel callers by decreasing the FPR for both SNP and indel calling and by increasing the TPR for indel calling (Figure 2; Table S15). These results suggest that Coval-Refine is consistently effective in improving alignment in any organisms.

### Coval Improves ‘Targeted’ Alignments

**Figure 3:**
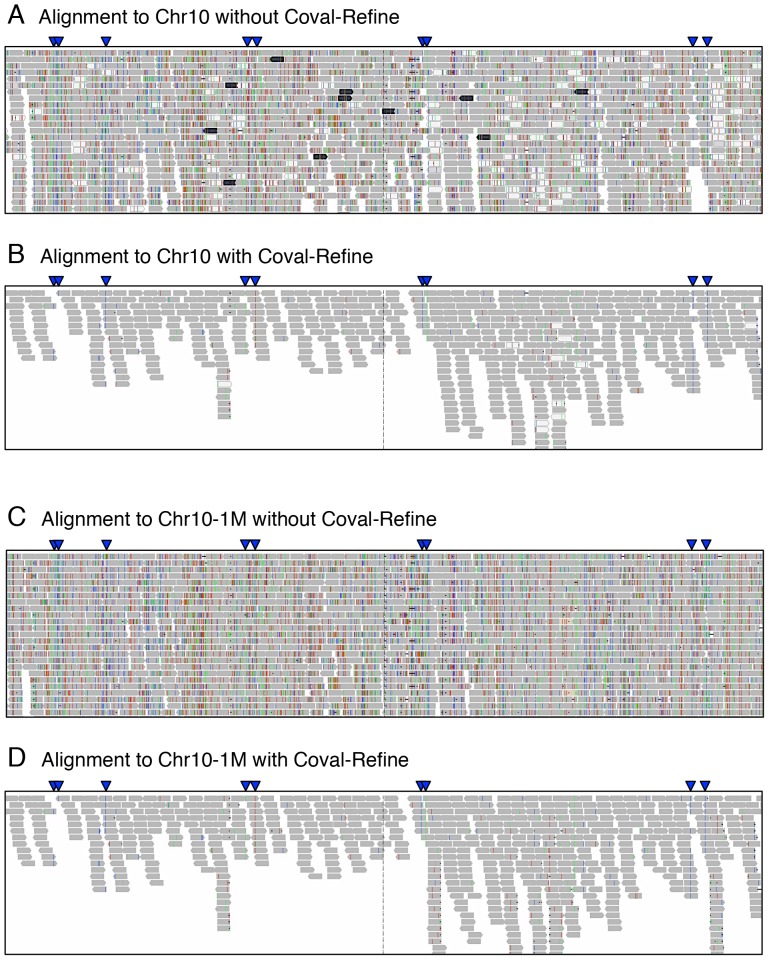
Improvement of targeted alignment by Coval-Refine. Rice whole-genome sequencing reads (63 million 75 bp paired-end reads) were aligned to chromosome 10 (A and B) or a 1 Mb region of chromosome 10 (C and D) of the simulated rice genome. Snapshot views of the alignments, corresponding to positions 1,338,000 to 1,342,538, with (B and D) or without (A and C) the Coval-Refine tool (basic mode) are represented. The shown alignment views were obtained with an IGV 1.5 viewer [46]. Shaded bars represent reads, and colored lines in bars non-reference bases. Blue arrowheads indicate true positive SNPs that had been introduced into the rice genome using the Coval-Simulate tool.

**Figure 4:**
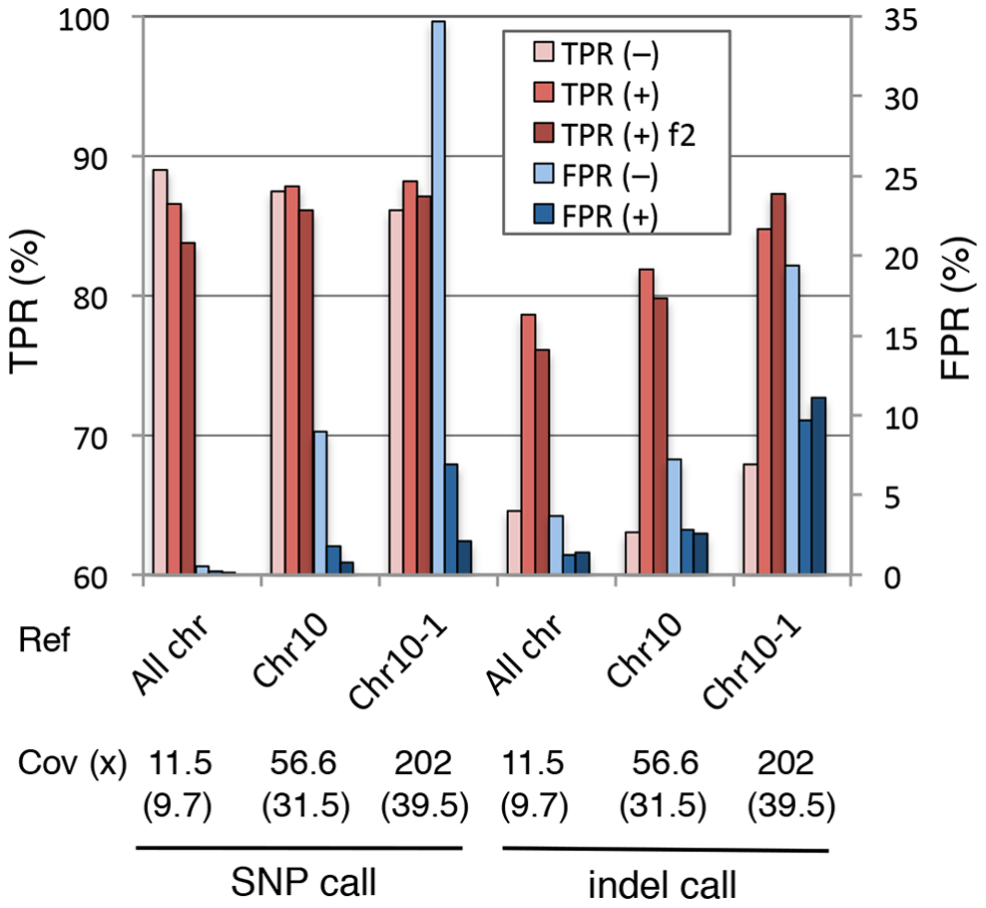
Improvement of SNP/indel calling accuracy by Coval-Refine in targeted alignment. The whole chromosomes (All chr), chromosome 10 (Chr10), a 1 Mb fragment of chromosome 10 (Chr10-1M: positions 1000001 to 2000000 of Chr10) from the simulated rice genome were aligned with 75-bp paired-end reads sequenced from the whole rice genome using BWA. The alignments were filtered (+, bars in dark- and middle-red and in dark- and middle-blue) or not filtered (–, bars in light red and in light blue) with Coval-Refine in the basic mode. Two different filtering conditions of Coval-Refine for mismatch reads were applied; one is the default option for removing reads with three or more mismatches (middle-red and middle-blue bars), the other removing the second paired-end mate read when the first mate is filtered and removing a read pair that contained more than two total mismatches (dark red and dark blue bars). The mean coverage of read depth before and after (indicated with parentheses) the Coval-Refine treatment is indicated under the reference chromosome name. Homozygous SNPs and indels were called as in Figure 1. TPR and FPR for the called SNPs are shown with red and blue bars, respectively.

Alignment of a local DNA fragment (*e.g.*, an assembled contig or a sequence from a DNA clone) with reads from whole genome sequencing or RNA-Seq is a common genomics task. When we carried out this kind of targeted alignment, we observed alignments with an extraordinary depth of coverage. To examine whether our method is effective in improving the quality of the targeted alignments, we conducted an *in silico* experiment using local rice genomic regions as simulated references. The 63 million rice paired-end reads sequenced from the rice whole genome were aligned to the entire chromosome 10 (chr10; 23.7 Mb) or a 1 Mb segment (chr10-1M) corresponding to chr10∶1000001–2000000 from the rice genome that contains artificial mutations. The alignment data for chr10 and chr10-1M had 4.9- and 17.6-fold higher coverage of depth, respectively, than that for the whole genome (Figure 3A, Figure S10), indicating that these local genomic regions were wrongly aligned, with many reads that should have been originally aligned to other chromosomal regions. We found that these wrongly aligned reads contained many more mismatches than reads from the whole-genome alignment (Table 3; Figure S10). When the alignments were filtered with Coval-Refine, they were remarkably refined due to removal of many misaligned reads (Figure 3) and the overall calling accuracy of SNPs and indels was significantly improved (Figure 4). The FPR was further decreased by removing the second mate of filtered paired-end reads when the first mate was filtered out and by removing read pairs that contained more than two total mismatches. Although filtering based on mapping quality had some effects on decreasing misaligned reads in the targeted alignments, these effects were substantially smaller than the filtering effects of Coval (Table S17). These results indicate that our method can effectively improve poor alignment data containing misaligned reads as well as reads containing multiple sequencing errors.

**Table 3:** Increased mismatches of reads observed in a targeted alignment.

Minimal number of mismatch	Content of reads (%)	
	Whole alignment (chr1-chr12)	Targeted alignment (chr10)
1	14.6	59.3
2	6.1	40.2
3	3.7	27.8
4	2.3	18.2
5	1.3	10.1

Rice whole genome sequencing reads were aligned to the rice whole genome or chromosome 10 alone. Among the concordantly aligned paired-end reads, fractions of reads greater than or equal to the indicated number of mismatches were calculate.

## Discussion

The clustered high-mismatch reads observed in alignments with Illumina reads are likely due to sequence-specific errors stemming from the Illumina sequencing system [2]. Because sequence reads containing substantial numbers of errors introduced through this mechanism cannot be aligned to the reference sequence, the error-prone genomic regions tend to have lower read coverage, containing high-mismatch reads that are still tolerated for the alignment. Thus, alignment regions with low coverage have a high potential for miscalling DNA variants, owing to both low read coverage and a high number of mismatch-containing reads within these regions. Although gap-free aligners tend not to align such high-mismatch reads, these aligners have the disadvantages of poor performance in detection of indels and SNP calling accuracy, as shown in Figure S7 and as described previously [13]. Thus, our method could be particularly useful for calling variants in such error-prone regions due to possible sequence-specific errors.

The fact that the sequence-specific incidence of sequencing errors is still unpredictable makes it difficult to generate artificial simulated reads with error properties resembling natural reads from real genomes. Thus, simulation experiments with artificial reads may not faithfully evaluate the performance of DNA variant callers. On the other hand, performance evaluation using real variant data, such as dbSNP and array data, allows us to determine sensitivity for variant calling, but not specificity. The simulation strategy, using both simulated genomic data and real read data, could solve these problems. By computationally introducing SNPs and indels into a reference genome sequence, we can use real short-read sequences to assess various filtering parameters. To conduct the simulation analysis with real reads, pre-existing DNA variants observed between a reference sequence and sequencing reads must be removed. Complete genome sequences, even those of inbred model organisms such as nematode, *Arabidopsis*, and rice, exhibit differences in sequence between individuals maintained in different regions or laboratories, probably due to accumulated spontaneous mutations and chromosomal rearrangements [44], [45], as well as to intrinsic sequencing errors in public reference sequences. This leads to the problem of classification of substantial numbers of SNPs and indels as false positives in a simulation test. Although the substitution step of endogenous variants involves the replacement of a small number of false-positive bases, which could also decrease real (error-derived) false positives in subsequent simulation tests, this would be minimized by substituting endogenous DNA variants selected using aligners and reads that are different from those used for the simulation tests (Table S2).

Our filtering method copes not only with sequencing errors but also with alignment errors. When whole-genome sequencing reads are aligned to a local chromosome region, we have observed many wrongly mapped reads that should have been aligned to other regions. This causes an alignment with a much higher depth of read coverage than a normal whole genome alignment, as shown in Figure 3. These wrongly aligned reads tend to have a higher number of mismatches against the reference than truly aligned reads. Coval can improve such poor alignments by filtering potentially misaligned reads with multiple mismatches. ‘Targeted’ alignments using reads from whole-genome sequencing, RNA-Seq, or metagenomic sequencing have been performed in many cases, and sequencing from libraries containing contaminated genomes or mRNA increases the chance of misalignment. Coval could be particularly useful when conducting alignment experiments with sequences such as this that are susceptible to misalignment.

Accurate calling of SNPs/indels by Coval is largely due to the strategy that eliminates and repairs poorly or wrongly aligned reads. This alignment improvement cannot be attained through a quality-based filtering strategy, such as pre-filtering or trimming of low-quality reads and filtering of aligned reads with low mapping quality. The Coval-Call component is not designed for calling DNA variants from exon-captured data, RNA-Seq data, pooled samples, or some specific samples such as DNA from human tumors. Therefore, other DNA variant callers that are designed for these specific purposes should be used, and pre-treatment of alignments with Coval-Refine could improve the performance of these other DNA variant callers. Moreover, the enhancement of alignments by Coval should be applicable to other alignment-based analyses, including RNA-Seq, Chip-Seq, and bisulfite sequencing.

## Supporting Information

Figure S1 **Outline of Coval pipeline and schematic description of Coval-Refine algorithm.**
(PDF)

Figure S2 **Algorithm of Coval-Refine error correction.**
(PDF)

Figure S3 **Snapshot view of Illumina short read alignments.**
(PDF)

Figure S4 **Base quality distribution of rice Illumina paired-end reads used for this study.**
(PDF)

Figure S5 **Abundance of high-mismatch reads in low to high read-depth regions.**
(PDF)

Figure S6 **Coval-Refine in ‘basic’ and ‘error correction’ modes.**
(PDF)

Figure S7 **SNP/indel calling performance of Coval for alignment data generated by different aligners.**
(PDF)

Figure S8 **SNP/indel calling from alignment data generated from different numbers of reads.**
(PDF)

Figure S9 **SNP/indel calling accuracy depending on threshold number of supporting reads.**
(PDF)

Figure S10 **Alignments targeted to local chromosomal regions have increased misaligned reads.**
(PDF)

Table S1 **Dataset used in this study.**
(PDF)

Table S2 **Simulation test with consensus references generated by different strategies.**
(PDF)

Table S3 **Commands and options of DNA variant callers used in this study.**
(PDF)

Table S4 **Number of mismatches in rice reads aligned with different alignment tools.**
(PDF)

Table S5 **SNP calling accuracy for different numbers of covered reads.**
(PDF)

Table S6 **SNP/indel calling accuracy for real (experimental) and simulated reads.**
(PDF)

Table S7 **SNP/indel calling accuracy for alignment using filtered/trimmed reads.**
(PDF)

Table S8 **Application of Coval to simulated rice genomes with different content of SNP.**
(PDF)

Table S9 **Application of Coval to simulated rice genomes with different content of indel.**
(PDF)

Table S10 **Call of clustered variants with Coval.**
(PDF)

Table S11 **Calling accuracy of heterozygous SNPs with simulated reads.**
(PDF)

Table S12 **Calling accuracy of heterozygous SNPs with experimentally obtained reads.**
(PDF)

Table S13 **Improvement of SNP calling accuracies of various SNP callers by Coval-Refine.**
(PDF)

Table S14 **Improvement of indel calling accuracies of various indel callers by Coval-Refine.**
(PDF)

Table S15 **Improvement by Coval-Refine of SNP/indel calling tools for mouse alignment data.**
(PDF)

Table S16 **SNP/indel calling accuracy of Coval for nematode alignment data.**
(PDF)

Table S17 **Effect of filtering with alignment mapping quality for SNP/indel calling from targeted alignment data.**
(PDF)
