# Correction: Coval: Improving Alignment Quality and Variant Calling Accuracy for Next-Generation Sequencing Data

**DOI:** 10.1371/annotation/cc88d2b5-36e8-441a-ab5f-58a9ed143d6b

**Published:** 2014-01-03

**Authors:** Shunichi Kosugi, Satoshi Natsume, Kentaro Yoshida, Daniel MacLean, Liliana Cano, Sophien Kamoun, Ryohei Terauchi

There is an error in the title for the last/fifth column of Table 2. "True positive rate" is incorrectly listed as the title for the last/fifth column. The correct title for this column is "False positive rate."

