# Supplementary material for: Coval: Improving Alignment Quality and Variant Calling Accuracy for Next-Generation Sequencing Data
Source: PLoS One. 2013 Oct 8;8(10):e75402. doi: 10.1371/journal.pone.0075402 (PMC3792961; doi:10.1371/journal.pone.0075402)
Supplement: Figure S1 — Outline of Coval pipeline and schematic description of Coval-Refine algorithm. (PDF) [file pone.0075402.s001.pdf]

Figure S1

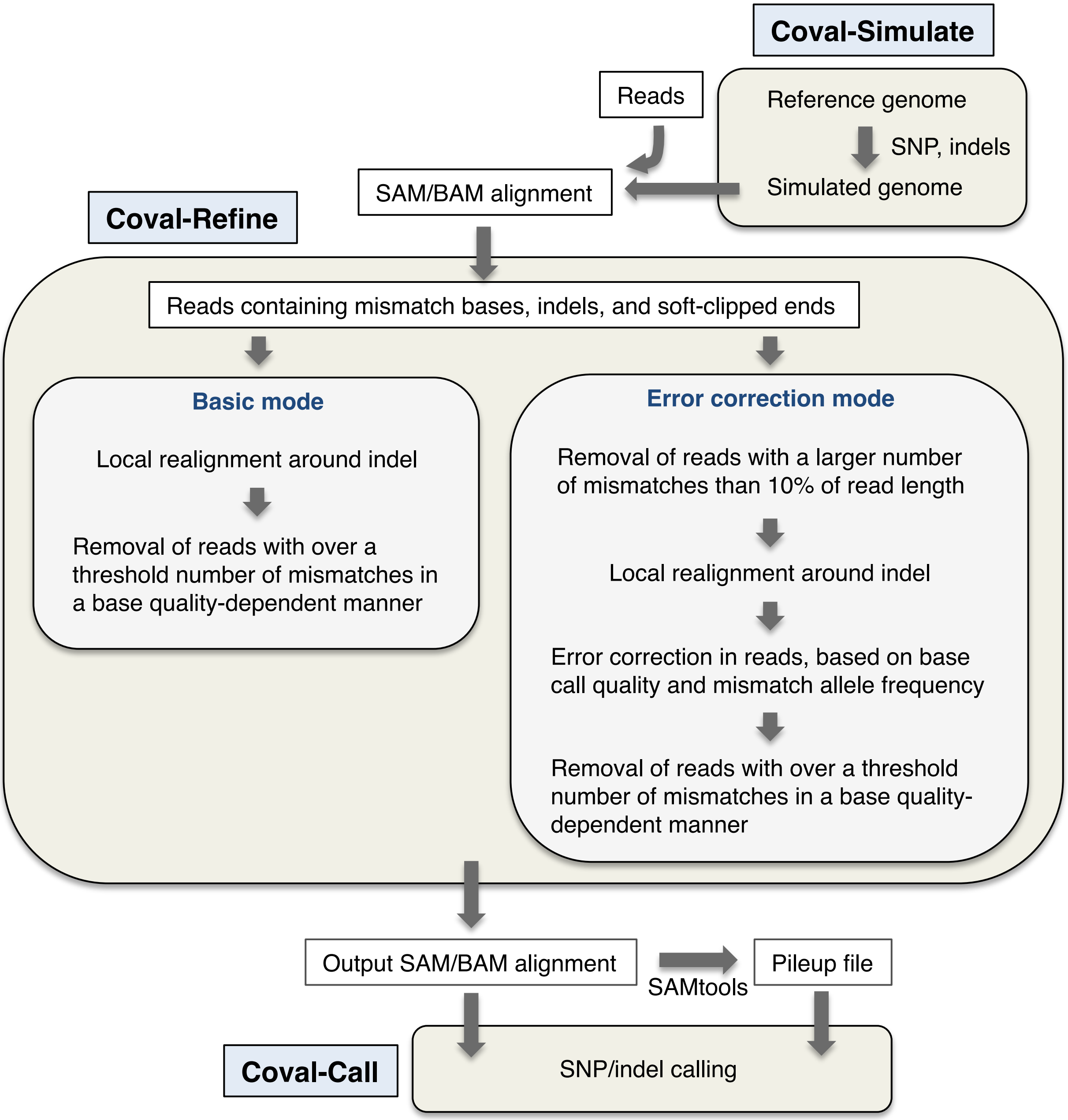

Figure S1. Outline of Coval pipeline and schematic description of Coval-Refine algorithm.

The quality of SAM/BAM alignment data is improved using the Coval-Refine component of the Coval pipeline, and the Coval- treated data are converted to a pileup file with SAMtools to call SNPs/indels using the Coval-Call component. To evaluate SNP/indel calling accuracy, artificial SNPs and/or indels can be introduced into a reference genome using the Coval-Simulate component to generate a simulated genome. The simulated genome is aligned with experimentally sequencing reads to obtain SAM/BAM data. Coval-Refine can be implemented mainly in two different modes, 'basic' and 'error correction'. In the basic mode, reads containing mismatches, indels, and a soft-clipped end are filtered to remove reads with over a given threshold number of mismatches after local realignment around indel. The filtering of mismatched reads is also controlled with the sum of mismatches contained in two paired reads and the base call quality of mismatch bases. In the error correction mode, reads with mismatches corresponding to over 10% of read length are filtered out before local realignment. After local realignment the reads are error-corrected based on the base call quality and allele frequency of mismatch bases, and then reads with over a given threshold number of mismatches are removed.
