# Supplementary material for: Coval: Improving Alignment Quality and Variant Calling Accuracy for Next-Generation Sequencing Data
Source: PLoS One. 2013 Oct 8;8(10):e75402. doi: 10.1371/journal.pone.0075402 (PMC3792961; doi:10.1371/journal.pone.0075402)
Supplement: Figure S2 — Algorithm of Coval-Refine error correction. (PDF) [file pone.0075402.s002.pdf]

Figure S2

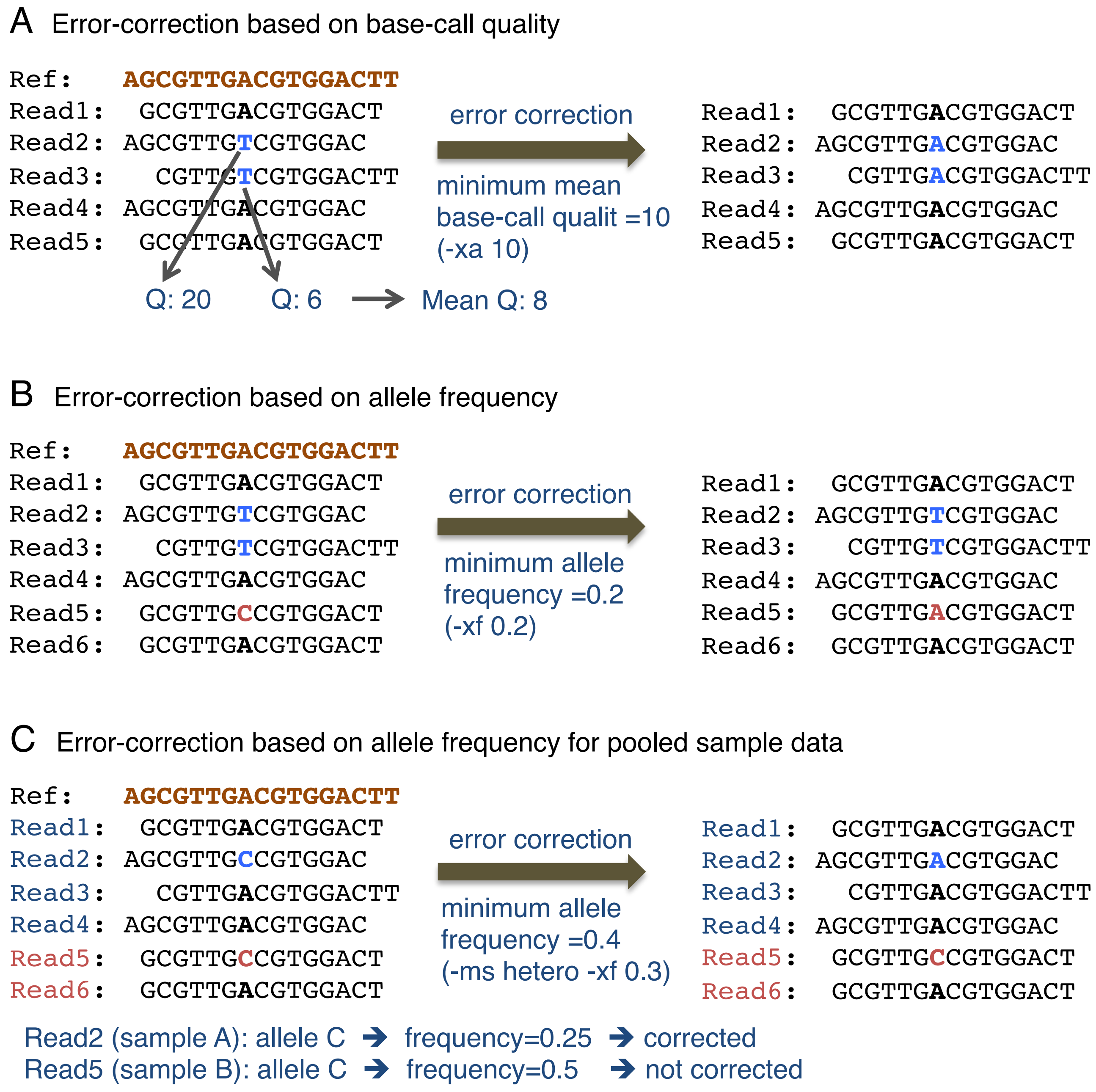

Figure S2. Algorithm of Coval-Refine error correction.

In the error correction mode of Coval-Refine, the error correction is conducted according to two criteria specified by user. One is based on base-call quality (A): correcting mismatch bases when the mean quality of non-reference bases supported at the site is lower than a minimum base quality (10 in this case), and the other based on allele frequency (B): correcting mismatch bases when the frequency of the non-reference allele is lower than a minimum allele frequency (0.2 in this case). The allele frequency-based correction for pooled sample data can be performed for reads of each sample by specifying –ms option (C), where read names of sample A are marked in blue, and those of sample B in red. Mismatch bases that meet either criterion are corrected to match the corresponding reference base.
