# Supplementary material for: Coval: Improving Alignment Quality and Variant Calling Accuracy for Next-Generation Sequencing Data
Source: PLoS One. 2013 Oct 8;8(10):e75402. doi: 10.1371/journal.pone.0075402 (PMC3792961; doi:10.1371/journal.pone.0075402)
Supplement: Figure S3 — Snapshot view of Illumina short read alignments. (PDF) [file pone.0075402.s003.pdf]

Figure S3

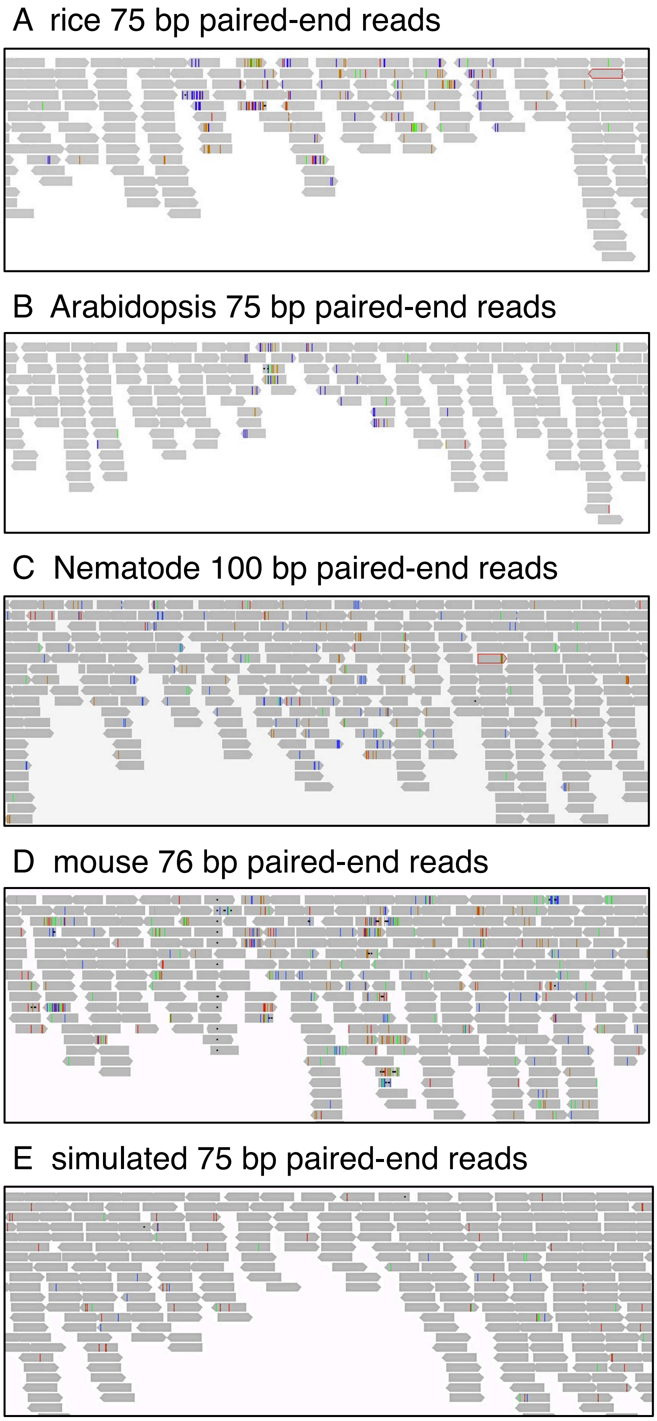

Figure S3. Snapshot view of Illumina short read alignments.

Paired-end reads of rice, *Arabidopsis*, nematode, and mouse, and artificial paired-end reads of rice were aligned to reference genomes of the corresponding species with the default option of BWA. The artificial simulated reads were generated by incorporating the error and read depth properties of the rice real reads using a pIRS simulator. The average read depth calculated for each alignment was 11.5× for rice (A), 15.2× for nematode (B), 11.8× for mouse (C), and 12.3× for simulated reads (D). The shown alignments are typical views, obtained with an IGV 1.5 viewer. Shaded bars represent reads, and colored lines in bars non-reference bases.
