# Supplementary material for: Coval: Improving Alignment Quality and Variant Calling Accuracy for Next-Generation Sequencing Data
Source: PLoS One. 2013 Oct 8;8(10):e75402. doi: 10.1371/journal.pone.0075402 (PMC3792961; doi:10.1371/journal.pone.0075402)
Supplement: Figure S4 — Base quality distribution of rice Illumina paired-end reads used for this study. (PDF) [file pone.0075402.s004.pdf]

Figure S4

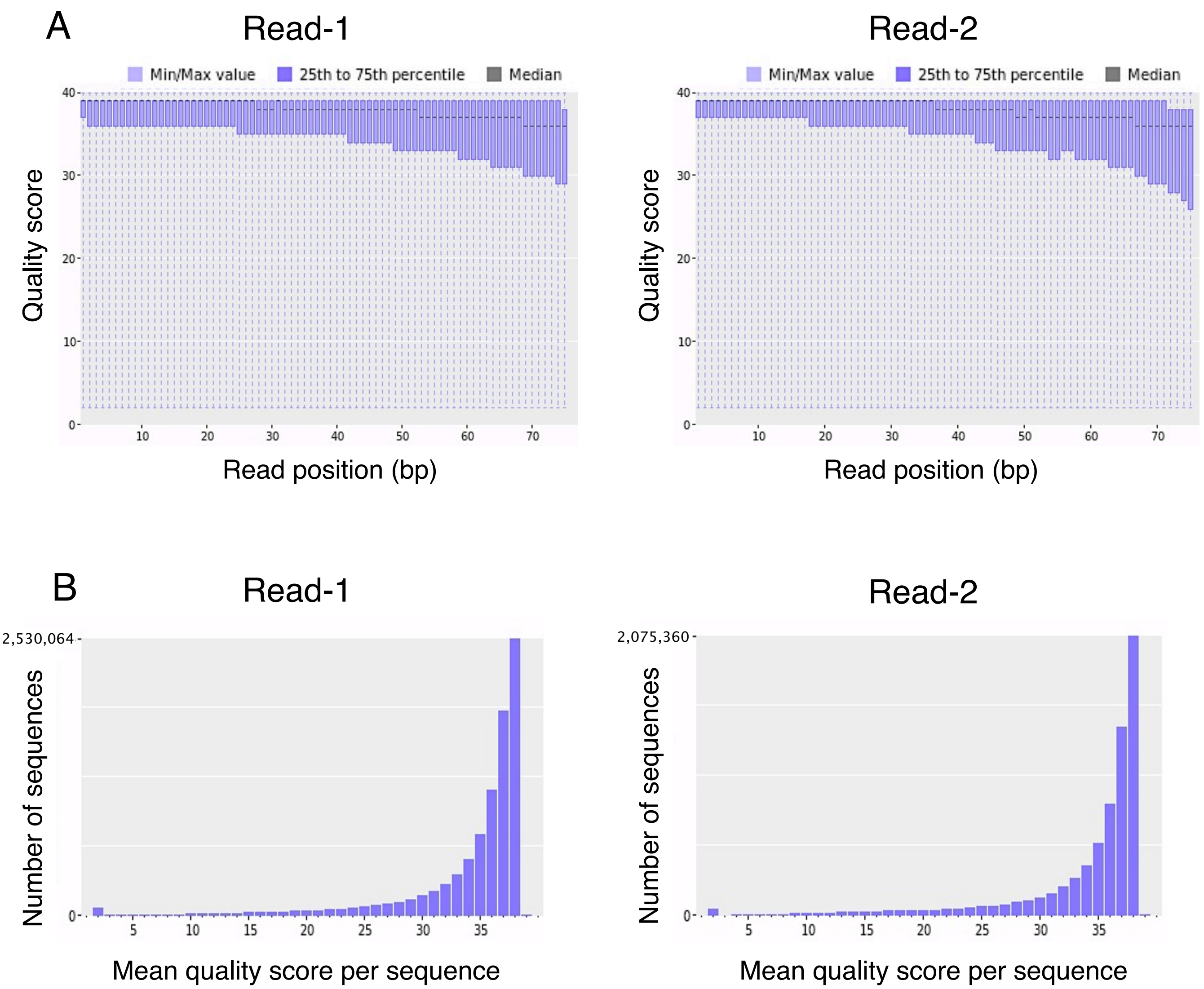

Figure S4. Base quality distribution of rice Illumina paired-end reads used for this study.

(A) Distribution and median of Phred base quality scores of rice reads at each read position. Statistical analysis of base quality distribution for each pair (Read1 and Read2) of 31.5 million rice Illumina paired-end reads was carried out through the PRINSEQ web service (http://edwards.sdsu.edu/prinseq_beta/). The indicated illustrations are snapshots clipped from PRINSEQ outputs displayed on a browser. (B) Number of reads exhibiting each mean quality score (1 to 40) per sequence.
