# Supplementary material for: Coval: Improving Alignment Quality and Variant Calling Accuracy for Next-Generation Sequencing Data
Source: PLoS One. 2013 Oct 8;8(10):e75402. doi: 10.1371/journal.pone.0075402 (PMC3792961; doi:10.1371/journal.pone.0075402)
Supplement: Figure S5 — Abundance of high-mismatch reads in low to high read-depth regions. (PDF) [file pone.0075402.s005.pdf]

Figure S5

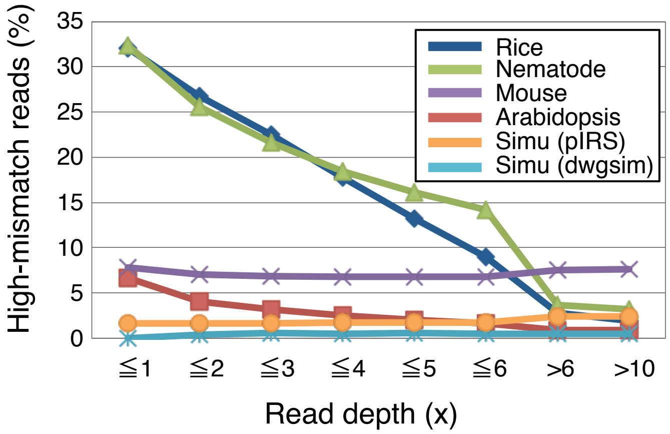

Figure S5. Abundance of high-mismatch reads in low to high read-depth regions.

Paired-end reads of the indicated species or artificial simulated reads were aligned to the corresponding reference genomes using BWA. Simulated reads (Simu-dwgsim and Simu-pirs) were generated using the dwgsim and pIRS simulation tools, respectively. Using the alignment data, we calculated the average percentage of reads with at least three mismatches (high-mismatch reads) in each 150 bp (200 bp for nematode) window for the indicated read depth. The average read depths for the alignments were: 11.5× for rice, 10.5× for *Arabidopsis*, 15.2× for nematode, 11.8× for mouse, 12.3× for dwgsim-simulated reads, and 12.3× for pIRS-simulated reads.
