# Supplementary material for: Coval: Improving Alignment Quality and Variant Calling Accuracy for Next-Generation Sequencing Data
Source: PLoS One. 2013 Oct 8;8(10):e75402. doi: 10.1371/journal.pone.0075402 (PMC3792961; doi:10.1371/journal.pone.0075402)
Supplement: Figure S6 — Coval-Refine in ‘basic’ and ‘error correction’ modes. (PDF) [file pone.0075402.s006.pdf]

Figure S6

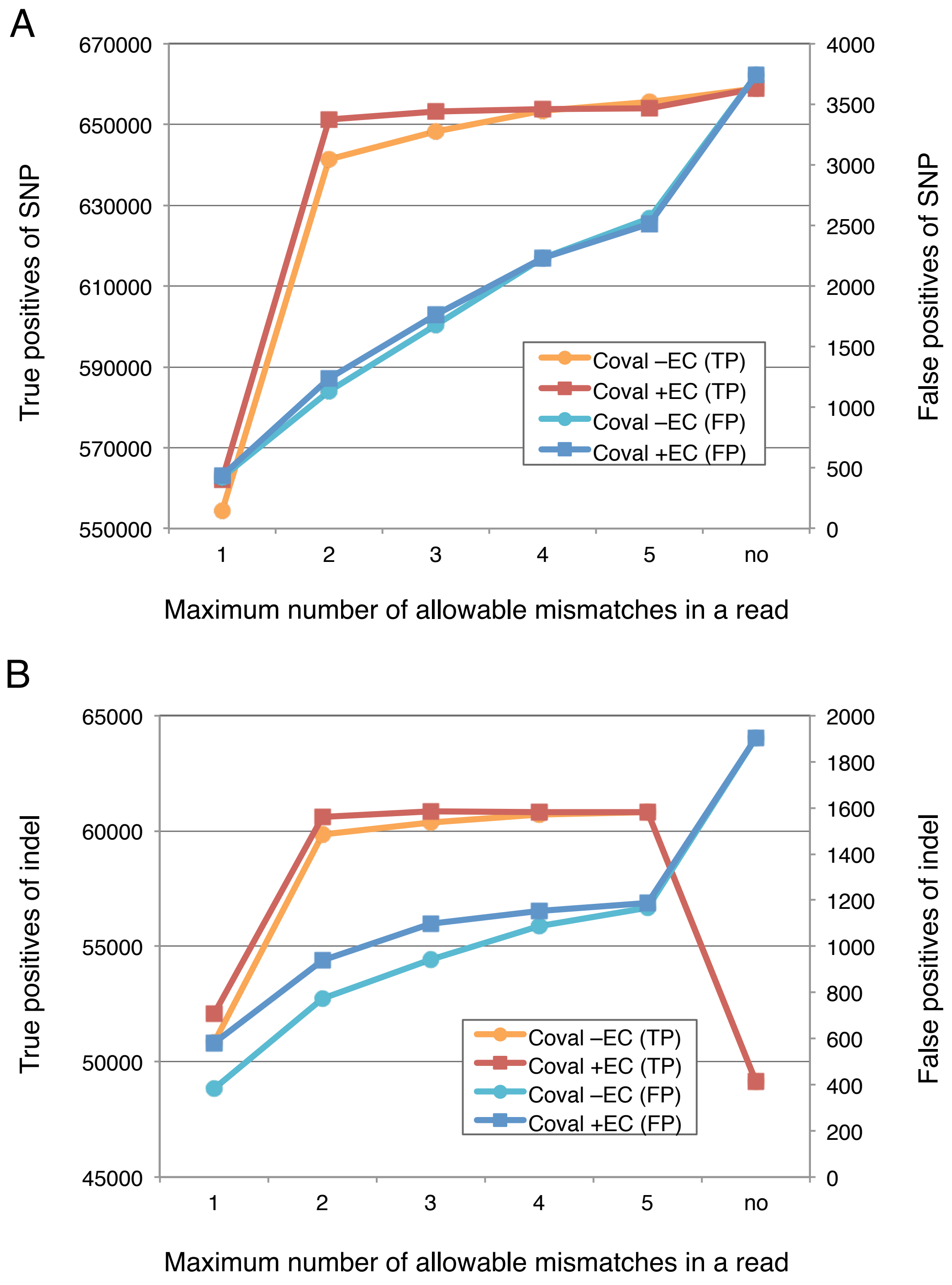

Figure S6. Coval-Refine in 'basic' and 'error correction' modes.

(A) SNP calling accuracy with or without Coval-Refine. (B) Indel calling accuracy with or without Coval-Refine. The simulated rice genome was aligned with the rice reads using BWA. The alignments were filtered with Coval-Refine in the basic mode (Coval –EC: orange and light blue lines with circles) and the error correction mode (Coval +EC: red and blue lines with squares). After removing reads containing mismatches that were greater than the number indicated on the x-axis with Coval-Refine, homozygous SNPs or indels were called using Coval-Call with "minimum allele frequency=0.8" and "minimum number of reads supporting non-reference allele=2". Results without the filtering correspond to 'no' on the x-axis. True positives and false positives for the called SNPs are shown with red/orange and blue/light-blue lines, respectively.
