# Supplementary material for: Coval: Improving Alignment Quality and Variant Calling Accuracy for Next-Generation Sequencing Data
Source: PLoS One. 2013 Oct 8;8(10):e75402. doi: 10.1371/journal.pone.0075402 (PMC3792961; doi:10.1371/journal.pone.0075402)
Supplement: Figure S7 — SNP/indel calling performance of Coval for alignment data generated by different aligners. (PDF) [file pone.0075402.s007.pdf]

Figure S7

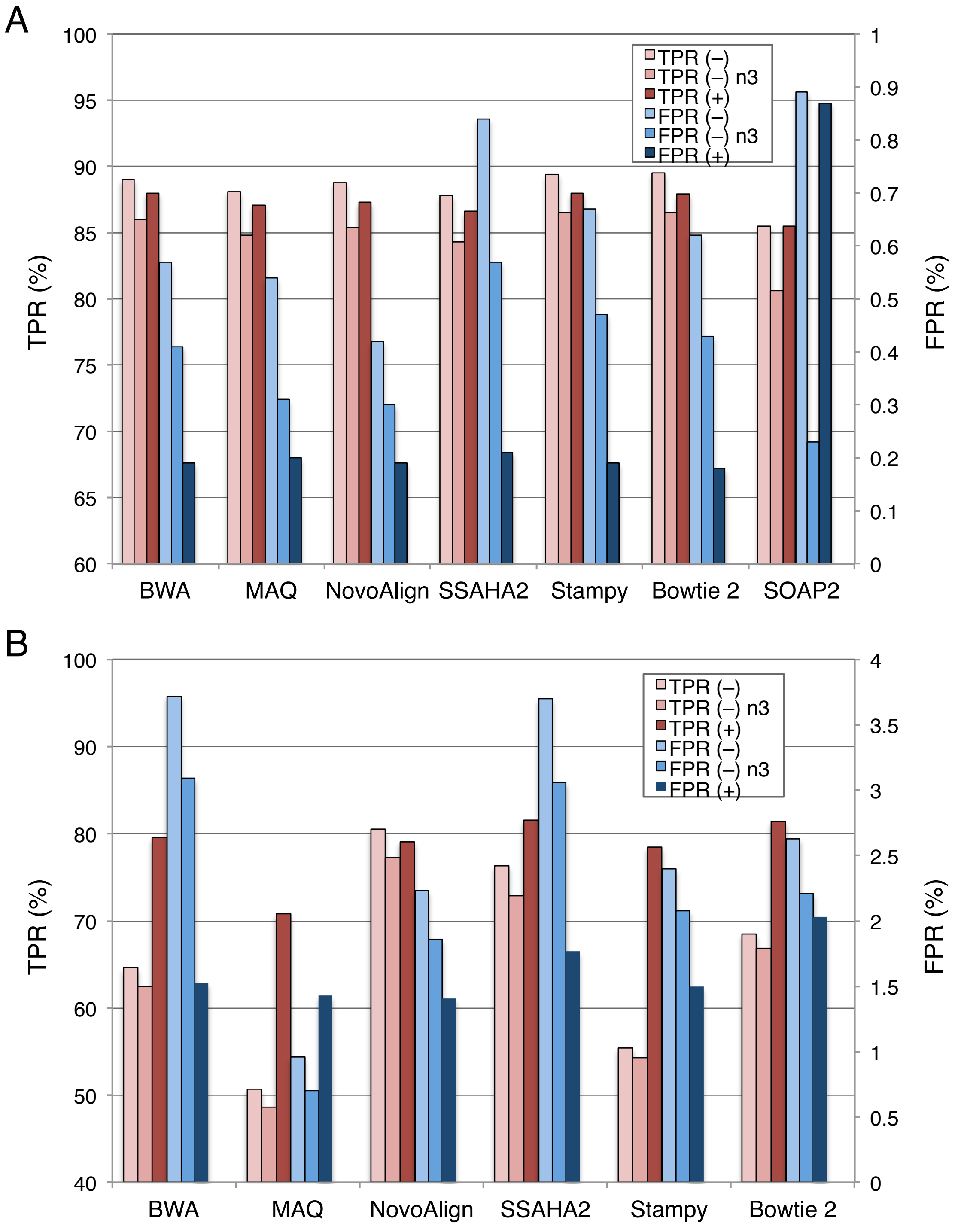

Figure S7. SNP/indel calling performance of Coval for alignment data generated by different aligners.

(A) SNP calling accuracy with or without Coval-Refine. (B) Indel calling accuracy with or without Coval-Refine. The simulated rice genome was aligned with the rice real (experimental) sequence data using the indicated alignment tools. The alignments were filtered (+, bars in dark red and in dark blue) or not filtered (–, bars in light- and middle-red and in light- and middle-blue) with Coval-Refine in the error correction mode (except for Novoalign in the basic mode), and SNPs and indels were called using Coval-Call with “minimum allele frequency=0.8” and “minimum number of reads supporting non-reference allele=2”. For another calling condition (n3, bars in middle-red and in middle-blue), SNPs/indels were called with “minimum number of reads supporting non-reference allele=3”, and the same for the other options. TPR and FPR for the called SNPs are shown with red and blue bars, respectively.
