# Supplementary material for: Coval: Improving Alignment Quality and Variant Calling Accuracy for Next-Generation Sequencing Data
Source: PLoS One. 2013 Oct 8;8(10):e75402. doi: 10.1371/journal.pone.0075402 (PMC3792961; doi:10.1371/journal.pone.0075402)
Supplement: Figure S8 — SNP/indel calling from alignment data generated from different numbers of reads. (PDF) [file pone.0075402.s008.pdf]

Figure S8

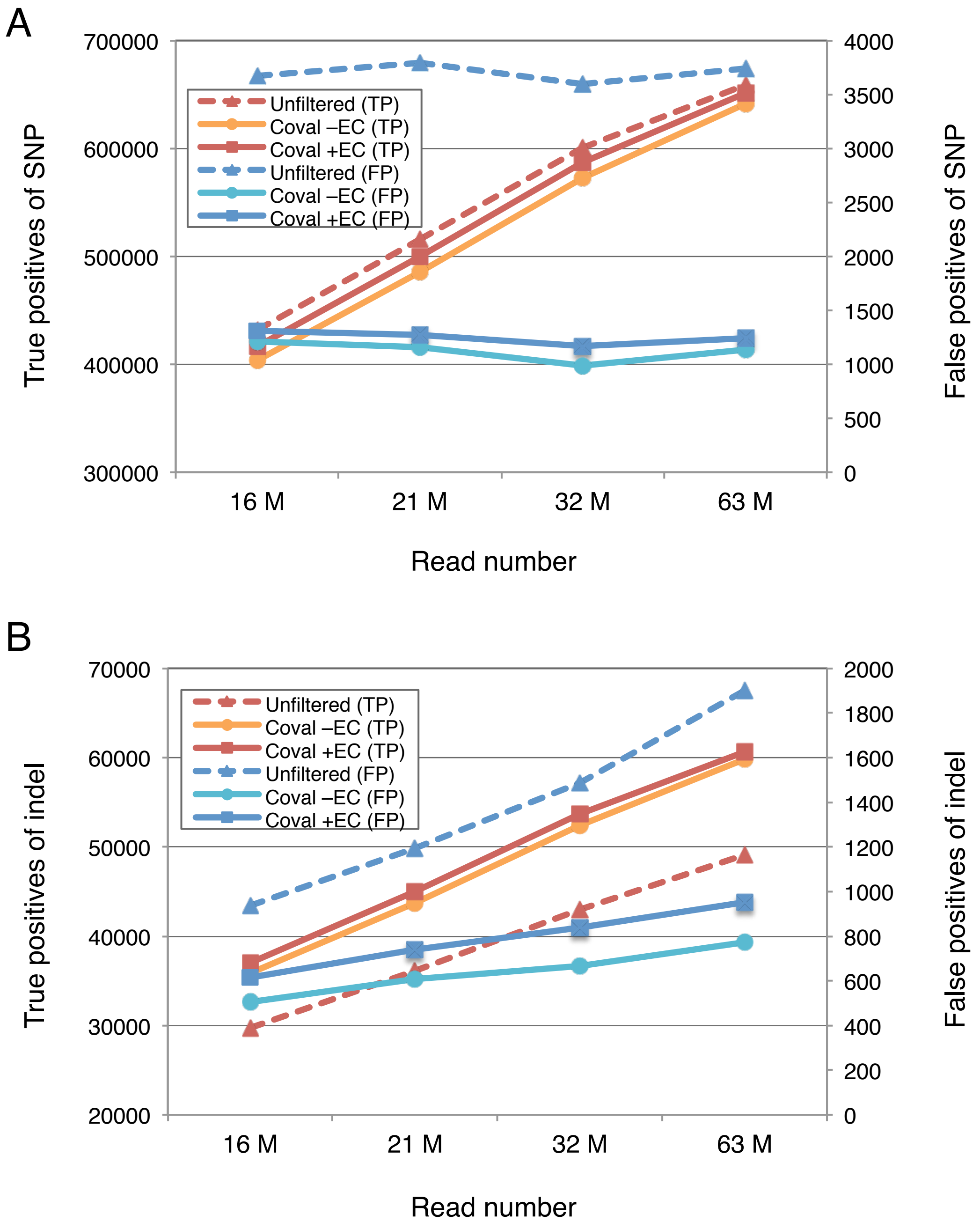

Figure S8. SNP/indel calling from alignment data generated from different numbers of reads.

(A) SNP calling accuracy with or without Coval-Refine. (B) Indel calling accuracy with or without Coval-Refine. The simulated rice genome was aligned with the indicated number of rice reads and filtered (solid lines) or not filtered (broken lines) with Coval-Refine in the basic mode (Coval –EC: orange and light blue lines with circles) and the error correction mode (Coval +EC: red and blue lines with squares) with "maximum number of allowable mismatches=2". Read depths of coverage of the alignments with16 M, 21 M, 32 M, and 63 M reads were 3x, 4.8x, 6x, and 11.5x, respectively. Homozygous SNPs or indels were called using Coval-Call with "minimum allele frequency=0.8" and "minimum number of reads supporting non-reference allele=2". True positives and false positives for the called SNPs are shown with red/orange and blue/light-blue lines, respectively.
