# Supplementary material for: Coval: Improving Alignment Quality and Variant Calling Accuracy for Next-Generation Sequencing Data
Source: PLoS One. 2013 Oct 8;8(10):e75402. doi: 10.1371/journal.pone.0075402 (PMC3792961; doi:10.1371/journal.pone.0075402)
Supplement: Figure S9 — SNP/indel calling accuracy depending on threshold number of supporting reads. (PDF) [file pone.0075402.s009.pdf]

Figure S9

A

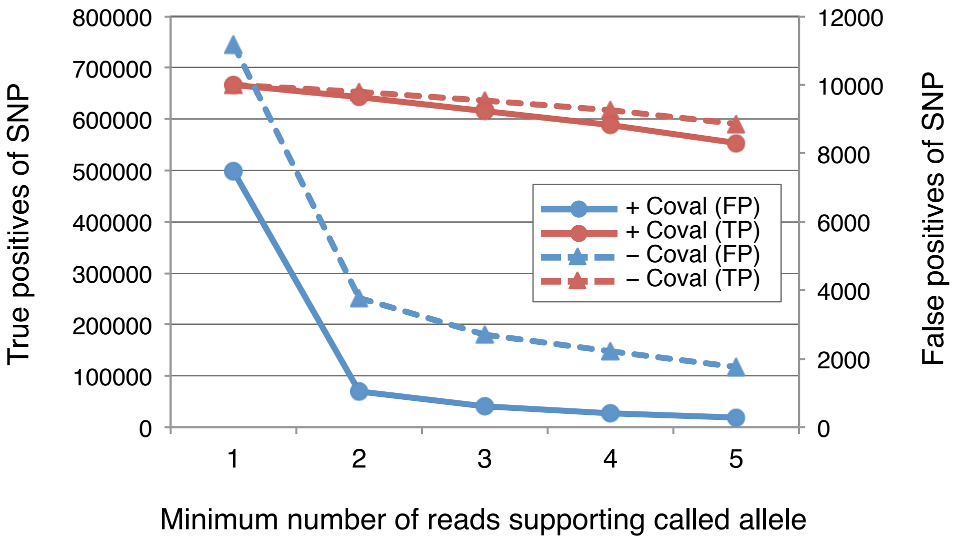

B

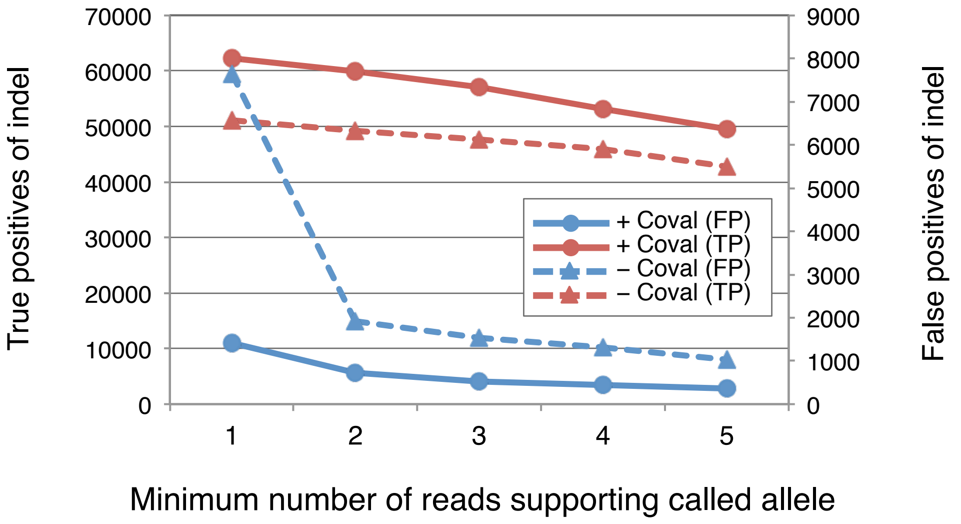

Figure S9. SNP/indel calling accuracy depending on threshold number of supporting reads.

(A) SNP calling accuracy with or without Coval-Refine. (B) Indel calling accuracy with or without Coval-Refine. SNPs and indels supported by the indicated minimum number of reads at the called allele positions were selected from the rice simulation data, as in Figure S4. The number of true positive (red lines) and false positive (blue lines) SNPs and indels were plotted on the y-axis. SNPs and indels were called from the data that had been treated with (solid lines) or without (broken lines) the Coval-Refine in the basic mode.
