# Supplementary material for: Coval: Improving Alignment Quality and Variant Calling Accuracy for Next-Generation Sequencing Data
Source: PLoS One. 2013 Oct 8;8(10):e75402. doi: 10.1371/journal.pone.0075402 (PMC3792961; doi:10.1371/journal.pone.0075402)
Supplement: Figure S10 — Alignments targeted to local chromosomal regions have increased misaligned reads. (PDF) [file pone.0075402.s010.pdf]

Figure S10

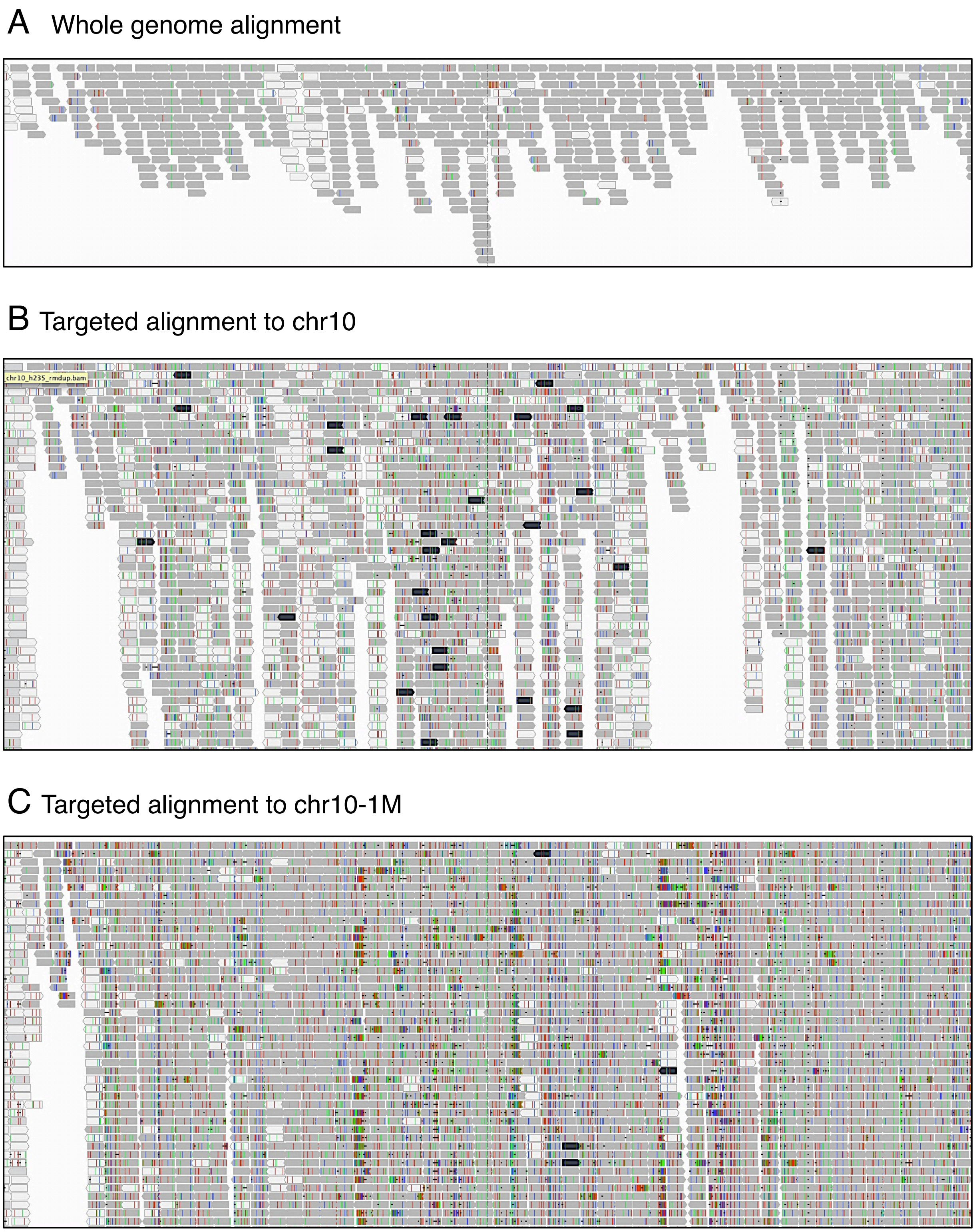

Figure S10. Alignments targeted to local chromosomal regions have increased misaligned reads.

Rice whole-genome sequencing reads (63 million 75 bp paired-end reads) were aligned to the rice whole genome (A), the rice chromosome 10 (B), and a 1 Mb region of the rice chromosome 10, corresponding to positions 1,000,001 to 2,000,000 (C). The shown alignments are typical views, obtained with an IGV 1.5 viewer, all of which are from an identical region of chromosome 10 corresponding to positions 1,257,001 to 1,261,528. Bars represent 75 bp reads, of which white bars represent reads with a mapping quality of 0 and black bars discordant paired reads with an unconventional insert size. Colored lines in bars indicate non-reference bases.
