# Supplementary material for: Coval: Improving Alignment Quality and Variant Calling Accuracy for Next-Generation Sequencing Data
Source: PLoS One. 2013 Oct 8;8(10):e75402. doi: 10.1371/journal.pone.0075402 (PMC3792961; doi:10.1371/journal.pone.0075402)
Supplement: Table S1 — Dataset used in this study. (PDF) [file pone.0075402.s011.pdf]

**Table S1. Dataset used in this study.**

| Species | Ref/Reads | Name/Accession number | Download (data deposited) site |
| --- | --- | --- | --- |
| Rice | Reference | IRGSP build 5 | RAP_BD (http://rapdb.dna.affrc.go.jp/) |
| | Reads | DRA000470 | DDBJ (http://trace.ddbj.nig.ac.jp/) |
| Arabidopsis | Reference | TAIR10 | TAIR (ftp://ftp.arabidopsis.org/home/tair/Sequences/) |
| | Reads | DRR013348 | DDBJ (http://trace.ddbj.nig.ac.jp/) |
| *C. elegans* | Reference | WS220 | NCBI (ftp://ftp.ncbi.nlm.nih.gov/genomes/) |
| | Reads | SRR065388 | NCBI (http://www.ncbi.nlm.nih.gov/Traces/sra/) |
| Mouse | Reference | 129S5/SvEvBrd | Wellcome Trust Sanger Institute (http://www.sanger.ac.uk/resources/mouse/genomes) |
| | Reads | ERR007818 ~ERR007841 | NCBI (http://www.ncbi.nlm.nih.gov/Traces/sra/) |
