# Supplementary material for: Coval: Improving Alignment Quality and Variant Calling Accuracy for Next-Generation Sequencing Data
Source: PLoS One. 2013 Oct 8;8(10):e75402. doi: 10.1371/journal.pone.0075402 (PMC3792961; doi:10.1371/journal.pone.0075402)
Supplement: Table S2 — Simulation test with consensus references generated by different strategies. (PDF) [file pone.0075402.s012.pdf]

Table S2. Simulation test with consensus references generated by different strategies.

Aligner for ref[a]	Read for ref[b]	Read for simu[c]	Coval-Refine	SNP calling accuracy	
				True positives	False positives
None[d]	None[d]	A	–	651,278 (88.0%)	6,565 (1.00%)
			+	640,890 (86.6%)	3,747 (0.58%)
BWA	B	B	–	631,675 (85.3%)	3,229 (0.51%)
			+	617,818 (83.4%)	765 (0.12%)
BWA	B	A	–	651,985 (88.0%)	3,790 (0.58%)
			+	641,460 (86.6%)	1,290 (0.20%)
BWA	A+B	A	–	651,633 (88.0%)	3,490 (0.53%)
			+	641,283 (86.6%)	961 (0.15%)
NovoAlign[e]	A+B	A	–	651,566 (88.0%)	3,647 (0.56%)
			+	641,292 (86.6%)	1,140 (0.18%)

Rice consensus genomes were generated by substituting reference bases with endogenous DNA variants that had been selected using different reads (the second column) and aligners (the first column), and used for the generation of simulated genomes for testing of SNP calling accuracy. The simulated genome was aligned with the indicated read set (the third column) using BWA. SNPs were called using Coval-Call with options “minimum allele frequency=0.8” and “minimum number of reads supporting non-reference allele=2” when either read A or read B was used, and with options “minimum allele frequency=0.9” and “minimum number of reads supporting non-reference allele=3” when both read A and read B were used.

[a] Alignment tools used for selection of endogenous DNA variants to generate consensus sequence.

[b] A different set (A and B) of 63 million Illumina paired-end reads, used for the selection of endogenous DNA variants to generate a consensus sequence.

[c] Reads used for alignment to detect simulated artificial variants.

[d] The rice reference genome was not modified; it was used directly for the generation of the simulated genome.

[e] Output option ‘–A 1’.
