# Supplementary material for: Coval: Improving Alignment Quality and Variant Calling Accuracy for Next-Generation Sequencing Data
Source: PLoS One. 2013 Oct 8;8(10):e75402. doi: 10.1371/journal.pone.0075402 (PMC3792961; doi:10.1371/journal.pone.0075402)
Supplement: Table S3 — Commands and options of DNA variant callers used in this study. (PDF) [file pone.0075402.s013.pdf]

Table S3. Commands and options of DNA variant callers used in this study.

Variant caller	Called variant	Command and option
Coval-Call	SNP	coval call –n 2 –f 0.8 –q 20 –m 35
SAMtools pileup/varFilter (v0.1.8)	SNP	samtools pileup –vcf<br>samtools.pl varFilter –d 2 –D 35 –S 10 –l 10 | awk '$4~/[ACGT]/&&$3!="*"&&$6>=20'
SAMtools mpileup/bcftools (v0.1.18)	SNP	samtools mpileup –uBf<br>bcftools view –vc –I 0.1 –t 0.002
Atlas-SNP2 (v1.4.1)	SNP	Atlas-SNP2.rb –y 2 –f 35 –s Illumina<br>Further filtered with awk '$6>1&&$6/$9>=0.8'
Varscan (v2.3.3)	SNP	VarScan.jar pileup2snp --min-coverage 2 --min-reads2 2 --min-avg-qual 20 --min-var-freq 0.8 --p-value 99e-02
GeMS (v1.0)	SNP	gems –d 1<br>Further filtered with awk '$3!=$4&&$5>=0.8'
GATK (v2.2-3)	SNP/indel	All the options were left default except for the following; '–cov ReadGroupCovariate', '–cov QualityScoreCovariate', '–cov CycleCovariate', and '–cov DinucCovariate' for CountCovariates and '–minindelCnt 2' for UnifiedGenotyper were used.
Coval-Call	Indel	coval call –n 2 –f 0.8 –m 35
SAMtools mpileup/bcftools (v0.1.18)	Indel	samtools mpileup –uf<br>bcftools view –vc –I 0.1 –t 0.002
Atlas-Indel2 (v1.4.1)	Indel	Atlas-Indel2.rb –I –t 2 –m 2 –v 0.8
VarScan (v2.2.8 or	Indel	VarScan.jar pileup2indel --min-coverage 2

v2.3.3) [a]		--min-reads2 2 --min-avg-qual 20 --min-var-freq 0.8 --p-value 99e-02
GATK (v2.2-3)	Indel	GenomeAnalysisTK.jar –T IndelGenotyperV2 --minCoverage 2 --minFraction 0.8 --minConsensusFraction 0.8 --minIndelCount 2 GenomeAnalysisTK.jar –T IndelRealigner –B:indels,VCF

[a] VarScan v2.3.3 was used for rice alignment data and v2.2.8 used for mouse data.
