# Supplementary material for: Coval: Improving Alignment Quality and Variant Calling Accuracy for Next-Generation Sequencing Data
Source: PLoS One. 2013 Oct 8;8(10):e75402. doi: 10.1371/journal.pone.0075402 (PMC3792961; doi:10.1371/journal.pone.0075402)
Supplement: Table S4 — Number of mismatches in rice reads aligned with different alignment tools. (PDF) [file pone.0075402.s014.pdf]

Table S4. Number of mismatches in rice reads aligned with different alignment tools.

Mismatch number	Percentage of mismatch reads in aligned reads (%) [a]				
	BWA	MAQ	NovoAlign	SOAP2	Bowtie [b]
1	8.6	8.5	8.8	3.4	10.5
2	2.7	2.6	2.6	1.1	4.3
3	1.6	0.78	1.4	0	0
4	1.2	0.49	0.89	0	0
5	0.4	0.35	0.59	0	0
>5	0.8	0.98	1.8	0	0

Sixty-three million Illumina paired-end reads of *O. sativa* cv. Nipponbare were aligned to the rice reference (IRGSP build 5) genome using the indicated alignment tools in a seeding mode, which allows at most two mismatches within a 20–30 bp seed of reads.

[a] The percentage of reads with the indicated number of mismatches out of the total number of rice reads aligned to the rice reference genome.

[b] Version 0.12.7, which is not the latest version (Bowtie 2).
