# Supplementary material for: Coval: Improving Alignment Quality and Variant Calling Accuracy for Next-Generation Sequencing Data
Source: PLoS One. 2013 Oct 8;8(10):e75402. doi: 10.1371/journal.pone.0075402 (PMC3792961; doi:10.1371/journal.pone.0075402)
Supplement: Table S5 — SNP calling accuracy for different numbers of covered reads. (PDF) [file pone.0075402.s015.pdf]

Table S5. SNP calling accuracy for different numbers of covered reads.

Aligned data	Read depth (x)	SNP calling accuracy [a]	
		True positive (TPR)	False positive (FPR)
Rice	2	15,975 (2.2%)	1,045 (6.1%)
	3	19,449 (2.6%)	860 (4.2%)
	4	25,468 (3.4%)	723 (2.8%)
	5	33,368 (4.5%)	651 (1.9%)
	>5	545,467 (73.7%)	5,591 (1.0%)
Nematode	2	3,260 (1.6%)	719 (18.1%)
	3	3,173 (1.6%)	229 (6.7%)
	4	4,470 (2.2%)	192 (4.1%)
	5	4,837 (2.4%)	146 (2.9%)
	>5	168,813 (84.5%)	998 (0.59%)

Simulated rice and nematode genomes, each containing artificial SNPs making up 0.2% of their genome size, were aligned with rice and nematode real reads, resulting in alignments with average read depth of 11.5× and 15.2×, respectively. To minimize the effect of read depth on SNP calling, two reads were randomly selected at each position that was covered by different numbers of reads. Homozygous non-reference alleles supported by the selected two reads were called as SNPs and assessed for concordance with the introduced SNP allele. The numbers of true and false positives of the called SNPs, along with the indicated number of covered reads (read depth) are shown in each column. The true positive rate (TPR), indicated in parentheses, is the percentage of true positives out of the total number of SNPs incorporated into the reference. The false positive rate (FPR) is the percentage of false positives out of the total number of called SNPs.

[a] SNPs called from two reads that were randomly selected from the covered reads at the called positions.
