# Supplementary material for: Coval: Improving Alignment Quality and Variant Calling Accuracy for Next-Generation Sequencing Data
Source: PLoS One. 2013 Oct 8;8(10):e75402. doi: 10.1371/journal.pone.0075402 (PMC3792961; doi:10.1371/journal.pone.0075402)
Supplement: Table S6 — SNP/indel calling accuracy for real (experimental) and simulated reads. (PDF) [file pone.0075402.s016.pdf]

Table S6. SNP/indel calling accuracy for real (experimental) and simulated reads.

Read	SNP/indel	SNP/indel calling accuracy	
		True positives rate	False positives rate
Real	SNP	658,735 (89.0%)	3,771 (0.57%)
	Indel	49,152 (64.6%)	1,901 (3.72%)
Simulated (dwgsim)	SNP	679,856 (91.8%)	198 (0.03%)
	Indel	54,777 (72.0%)	78 (0.14%)
Simulated (pIRS)	SNP	672,398 (90.8%)	404 (0.06%)
	Indel	53,574 (70.4%)	77 (0.14%)

Using BWA, 6.3×10^7 experimentally obtained 75 bp paired-end reads of Nipponbare (real) were aligned to the simulated rice Nipponbare reference genome containing 0.2% artificial SNPs and 0.02% artificial indels. The same number of artificial simulated reads (75 bp paired-end) were generated using dwgsim and pIRS simulation tools with the simulated rice genome as a template. The simulated reads were aligned to the wild-type Nipponbare reference sequence, which contained no artificial SNPs/indels. SNPs and indels were called using the Coval-Call tool with ≥ 0.8 of variant frequency and a minimum of two supported reads at the called site.
