# Supplementary material for: Coval: Improving Alignment Quality and Variant Calling Accuracy for Next-Generation Sequencing Data
Source: PLoS One. 2013 Oct 8;8(10):e75402. doi: 10.1371/journal.pone.0075402 (PMC3792961; doi:10.1371/journal.pone.0075402)
Supplement: Table S7 — SNP/indel calling accuracy for alignment using filtered/trimmed reads. (PDF) [file pone.0075402.s017.pdf]

Table S7. SNP/indel calling accuracy for alignment using filtered/trimmed reads.

Read pretreatment	Removed bases (%)	Variant called	True positives rate	False positives rate
Untreated	0	SNP	658,899 (89.0%)	3,744 (0.57%)
Trimmo q10	8.6		649,734 (87.8%)	3,660 (0.56%)
Trimmo q15	13.3		642,477 (86.8%)	3,515 (0.54%)
Trimmo q20	21.6		627,393 (84.7%)	3,330 (0.53%)
Condetri q5	13.3		616,184 (87.3%)	3,648 (0.56%)
Condetri q10	16.4		641,995 (86.7%)	3,537 (0.55%)
Untreated	0	Indel	49,152 (64.6%)	1,901 (3.72%)
Trimmo q10	8.6		47,903 (63.0%)	1,672 (3.37%)
Trimmo q15	13.3		47,111 (61.9%)	1,551 (3.19%)
Trimmo q20	21.6		45,467 (59.8%)	1,387 (2.96%)
Condetri q5	13.3		47,157 (62.0%)	1,632 (3.35%)
Condetri q10	16.4		46,605 (61.2%)	1,575 (3.27%)

Low-quality reads and the 3' low-quality regions of rice 75 bp paired-end reads (7.5×10^7) were filtered and trimmed with two available tools, Trimmomatic and Condetri, with options specified to yield several levels of quality. Trimmo q10, q15, and q20 represent reads trimmed using Trimmomatic with the option SLIDINGWINDOW:2:10, :2:15, and :2:20, respectively. Condeteri q5 and q10 represent reads trimmed using Condetri with the options –lq 5 and 10, respectively. The other commonly specified options were as follows; 'ILLUMINACLIP:GAII:adapter.fa: 1:40:15 LEADING:3 TRAILING:3 MINLEN:50' for Trimmomatic; and '–minlen=35 –ml=5 –lq=5' for Condeteri. The percentages of the total bases removed from the original reads are indicated in the second row. The filtered/trimmed reads were aligned to a rice simulated reference genome containing 0.2% homozygous SNPs and 0.02% homozygous indels. Homozygous SNPs and indels were called with the Coval-Call tool with ≥ 0.8 of variant frequency and a minimum of two supported reads at the called site.
