# Supplementary material for: Coval: Improving Alignment Quality and Variant Calling Accuracy for Next-Generation Sequencing Data
Source: PLoS One. 2013 Oct 8;8(10):e75402. doi: 10.1371/journal.pone.0075402 (PMC3792961; doi:10.1371/journal.pone.0075402)
Supplement: Table S8 — Application of Coval to simulated rice genomes with different content of SNP. (PDF) [file pone.0075402.s018.pdf]

Table S8. Application of Coval to simulated rice genomes with different content of SNP.

SNP content (%)	Coval-Filter	Maximum Mismatch number [a]	Variant calling accuracy	
			True positive rate	False positive rate
0.001	–	–	32,845 (88.6%)	3,390 (9.36%)
	+	1	31,655 (85.4%)	368 (1.15%)
	+	2	32,478 (87.7%)	947 (2.83%)
0.005	–	–	164,885 (89.0%)	3,460 (2.06%)
	+	1	155,377 (83.8%)	385 (0.25%)
	+	2	162,998 (87.9%)	988 (0.60%)
0.02	–	–	658,899 (89.0%)	3,744 (0.57%)
	+	1	562,184 (75.9%)	436 (0.077%)
	+	2	651,419 (88.0%)	1,244 (0.19%)
	+	3	653,118 (88.2%)	1,763 (0.27%)
0.5	–	–	1,644,446 (89.0%)	4,559 (0.28%)
	+	2	1,578,623 (85.4%)	1569 (0.10%)
	+	3	1,623,057 (87.8%)	2,203 (0.14%)
1.0	–	–	3,234,292 (87.7%)	5,501 (0.17%)
	+	2	2,831,202 (76.8%)	2,095 (0.074%)
	+	3	3,142,807 (85.3%)	2,874 (0.091%)
	+	4	3,223,686 (87.4%)	3,712 (0.12%)

The simulated rice genome containing the indicated rate of SNP and one-tenth of the indicated rate of indel relative to the rice whole genome (383 Mb) was aligned with the rice real sequence data using BWA. The alignments were filtered (+) or not filtered (–) with Coval-Refine in the error correction mode, and SNPs were called using Coval-Call with “minimum allele frequency=0.8” and “minimum number of reads supporting non-reference allele=2”.

[a] Coval-Refine removes reads with mismatches larger than the indicated number.
