# Supplementary material for: Coval: Improving Alignment Quality and Variant Calling Accuracy for Next-Generation Sequencing Data
Source: PLoS One. 2013 Oct 8;8(10):e75402. doi: 10.1371/journal.pone.0075402 (PMC3792961; doi:10.1371/journal.pone.0075402)
Supplement: Table S9 — Application of Coval to simulated rice genomes with different content of indel. (PDF) [file pone.0075402.s019.pdf]

Table S9. Application of Coval to simulated rice genomes with different content of indel.

Indel content (%)	Coval-Filter	Maximum Mismatch number [a]	Variant calling accuracy	
			True positive rate	False positive rate
0.0001	–	–	2,491 (66.9%)	1,806 (42.0%)
	+	1	3,015 (81.0%)	625 (17.2%)
	+	2	3,090 (83.0%)	834 (21.3%)
0.0005	–	–	12,252 (66.1%)	1,821 (12.9%)
	+	1	14,559 (78.6%)	628 (4.14%)
	+	2	15,233 (82.2%)	857 (5.33%)
0.002	–	–	49,152 (64.6%)	1,901 (3.72%)
	+	1	52,063 (68.4%)	581 (1.10%)
	+	2	60,661 (79.7%)	953 (1.55%)
	+	3	60,835 (79.9%)	1097 (1.77%)
0.05	–	–	123,944 (66.9%)	2,114 (1.68%)
	+	2	146,736 (79.2%)	1,291 (0.87%)
	+	3	150,938 (81.5%)	1,453 (0.95%)
0.1	–	–	237,905 (64.2%)	2,611 (1.09%)
	+	2	263,930 (71.2%)	1,683 (0.63%)
	+	3	292,932 (79.0%)	2,091 (0.71%)
	+	4	300,058 (81.0%)	2,269 (0.75%)

The simulated rice genome containing the indicated rate of indel and ten-fold of the indicated rate of SNP relative to the rice whole genome (383 Mb) was aligned with the rice real sequence data using BWA. Indel calling conditions with Coval-Call are as in Table S8.

[a] Coval-Refine removes reads with mismatches larger than the indicated number.
