# Supplementary material for: Coval: Improving Alignment Quality and Variant Calling Accuracy for Next-Generation Sequencing Data
Source: PLoS One. 2013 Oct 8;8(10):e75402. doi: 10.1371/journal.pone.0075402 (PMC3792961; doi:10.1371/journal.pone.0075402)
Supplement: Table S10 — Call of clustered variants with Coval. (PDF) [file pone.0075402.s020.pdf]

Table S10. Call of clustered variants with Coval.

Variants called	Coval-Refine	Threshold number of mismatch [a]	Variant calling accuracy	
			True positive rate [clustered variants]	False positive rate [all variants]
SNP	–	–	20,656 (86.1%)	3,745 (0.57%)
	+	3 [b]	17,902 (74.6%)	1,162 (0.18%)
	+	4 [c]	20,234 (84.4%)	1,763 (0.27%)
	+	5 [d]	20,267 (84.5%)	1,807 (0.28%)
	+	6 [e]	20,434 (85.2%)	2,231 (0.34%)
Indel	–	–	1,560 (67.2%)	1,901 (3.72%)
	+	3 [b]	1,607 (69.2%)	958 (1.56%)
	+	4 [c]	1,823 (78.5%)	1,097 (1.77%)
	+	5 [d]	1,824 (78.5%)	1,098 (1.77%)
	+	6 [e]	1,831 (78.8%)	1,152 (1.86%)

Clustered variants were defined as variants that at least three variants reside within a range of 75 bp. The indicated false positives are the number of mis-called variants that do not match all the variants introduced into the simulated genome.

[a] Minimum number of mismatch contained in two paired-end reads. When the sum of mismatch contained in paired-end reads exceeds the value, the two reads are filtered out.

[b] Coval-Refine options specified: --num 2 --fnum 3 (default).

[c] Coval-Refine options specified: --num 2 --fnum 4.

[d] Coval-Refine options specified: --num 3 --fnum 5.

[e] Coval-Refine options specified: --num 3 --fnum 6.
