# Supplementary material for: Coval: Improving Alignment Quality and Variant Calling Accuracy for Next-Generation Sequencing Data
Source: PLoS One. 2013 Oct 8;8(10):e75402. doi: 10.1371/journal.pone.0075402 (PMC3792961; doi:10.1371/journal.pone.0075402)
Supplement: Table S11 — Calling accuracy of heterozygous SNPs with simulated reads. (PDF) [file pone.0075402.s021.pdf]

Table S11. Calling accuracy of heterozygous SNPs with simulated reads.

Simulator	Coval-Refine	SNP calling accuracy	
		True positive rate	False positive rate
dwgsim	–	649,339 (96.7%)	3,679 (0.56%)
	+	643,233 (95.8%)	291 (0.045%)
pIRS	–	676,372 (90.7%)	379 (0.056%)
	+	673,110 (90.3%)	149 (0.022%)

The wild-type Nipponbare rice reference was aligned using BWA with 120 million simulated 75 bp paired-end reads generated by dwgsim or pIRS simulators. The simulated reads contained heterozygous SNPs, homozygous short indels, and sequencing errors, corresponding to 0.2%, 0.02%, and 0.6% (0.38% for pIRS) of the rice genome, respectively. The alignment data were filtered (+) or not filtered (–) using the Coval-Refine tool in the basic mode with an option 'minimum mapping quality=1' and without local realignment. SNPs were called using Coval-Call with ≥ 0.2 of variant frequency, a minimum of two supported reads for homozygous SNPs, and a minimum of three supported reads for heterozygous SNPs.
