# Supplementary material for: Coval: Improving Alignment Quality and Variant Calling Accuracy for Next-Generation Sequencing Data
Source: PLoS One. 2013 Oct 8;8(10):e75402. doi: 10.1371/journal.pone.0075402 (PMC3792961; doi:10.1371/journal.pone.0075402)
Supplement: Table S12 — Calling accuracy of heterozygous SNPs with experimentally obtained reads. (PDF) [file pone.0075402.s022.pdf]

Table S12. Calling accuracy of heterozygous SNPs with experimentally obtained reads.

Heterozygosity	Coval Refine	SNP calling accuracy	
		True positive rate	True positive rate
(a) 50%	–	617,351 (83.4%)	37,535 (5.73%)
	+	574,953 (77.6%)	3,652 (0.63%)
(b) 25%	–	542,399 (73.3%)	21,198 (3.76%)
	+	501,843 (67.8%)	5,827 (1.15%)
(c) 12.5%	–	375,386 (50.7%)	7,242 (1.89%)
	+	345,716 (46.7%)	2,897 (0.83%)

The experimentally obtained rice reads (60, 30, and 15 millions) were mixed with the simulated 75 bp paired-end reads (60, 90, and 105 millions) generated by dwgsim with the rice simulated genome as template, respectively, yielding 120 millions of reads. The read mixtures were aligned to the rice simulated genome, resulting in alignments with average read depth of 24× and the indicated percentages of heterozygosity. The SNPs were called using Coval-Call with a maximum of 80 reads covering the called positions, a minimum allele frequency at the called position of 0.2 (a), 0.1 (b), or 0.05 (c), a minimum of two reads (b, c) or three reads (a) supporting the called allele.
