# Supplementary material for: Coval: Improving Alignment Quality and Variant Calling Accuracy for Next-Generation Sequencing Data
Source: PLoS One. 2013 Oct 8;8(10):e75402. doi: 10.1371/journal.pone.0075402 (PMC3792961; doi:10.1371/journal.pone.0075402)
Supplement: Table S13 — Improvement of SNP calling accuracies of various SNP callers by Coval-Refine. (PDF) [file pone.0075402.s023.pdf]

Table S13. Improvement of SNP calling accuracies of various SNP callers by Coval-Refine.

SNP caller	Coval-Refine	SNP calling accuracy	
		True positive rate	False positive rate
No caller [a]	–	691,846 (93.5%)	1,450,883 (67.7%)
Coval-Call	–	658,899 (89.0%)	3,744 (0.57%)
	+ [c]	641,333 (86.6%)	1,133 (0.18%)
	+ [d]	651,419 (88.0%)	1,244 (0.19%)
SAMtools pileup/ varFilter	–	597,087 (80.7%)	10,097 (1.66%)
	+ [c]	575,855 (77.8%)	1,394 (0.24%)
	+ [d]	591,188 (79.9%)	1,056 (0.18%)
SAMtools mpileup/ bcftools	–	646,269 (87.3%)	21,760 (3.26%)
	+ [c]	627,216 (84.7%)	2,419 (0.38%)
	+ [d]	621,238 (83.9%)	1,313 (0.21%)
Atlas-SNP2	–	642,449 (86.8%)	6,355 (0.98%)
	+ [c]	629,292 (85.0%)	2,467 (0.39%)
	+ [d]	617,156 (83.4%)	1122 (0.18%)
VarScan 2	–	664,809 (89.8%)	20,412 (2.98%)
	+ [c]	644,706 (87.1%)	3,307 (0.51%)
	+ [d]	652,778 (88.2%)	2592 (0.40%)
GATK	–	630,043 (85.1%)	12,752 (1.98%)
	+ [c]	614,276 (83.0%)	2,289 (0.37%)
	+ [d]	605,026 (81.7%)	1,596 (0.26%)
GATK [b] (+BQSR)	–	629,585 (85.0%)	4,378 (0.69%)
	+ [c]	612,619 (82.8%)	1,278 (0.21%)
	+ [d]	620,145 (83.8%)	1,265 (0.20%)
GeMS	–	635,999 (85.9%)	17,588 (2.69%)
	+ [c]	617,072 (83.4%)	3,284 (0.53%)
	+ [d]	625,588 (84.5%)	2,500 (0.40%)

The simulated rice genome was aligned with reads of the real rice genome (experimental reads) using BWA. Alignment data were filtered (+) or not filtered (–) with Coval-Refine in the basic or error correction mode, and homozygous SNPs were called using the indicated SNP callers. The SNPs extracted by all the callers were further filtered under the same conditions, with a minimum allele frequency at the called position of 0.8, a minimum of two reads supporting the called allele, and/or a maximum of 35 reads covering the called positions.

[a] Variants called only with 'samtools pileup –vcf' command.

[b] Base quality score recalibration was applied using the artificially introduced SNP set.

[c] Coval-Refine without error correction (basic mode).

[d] Coval-Refine with error correction (error correction mode).

[e] Coval-Refine without local realignment and error correction.
