# Supplementary material for: Coval: Improving Alignment Quality and Variant Calling Accuracy for Next-Generation Sequencing Data
Source: PLoS One. 2013 Oct 8;8(10):e75402. doi: 10.1371/journal.pone.0075402 (PMC3792961; doi:10.1371/journal.pone.0075402)
Supplement: Table S14 — Improvement of indel calling accuracies of various indel callers by Coval-Refine. (PDF) [file pone.0075402.s024.pdf]

Table S14. Improvement of indel calling accuracies of various indel callers by Coval-Refine.

SNP caller	Coval-Refine	Indel calling accuracy	
		True positive rate	False positive rate
No caller [a]	–	65,334 (85.9%)	129,929 (66.5%)
Coval-Call	–	49,152 (64.6%)	1,901 (3.72%)
	+ [c]	59,831 (78.6%)	773 (1.28%)
	+ [d]	60,661 (79.7%)	953 (1.55%)
SAMtools mpileup/ bcftools	–	60,595 (79.6%)	1,272 (2.05%)
	+ [c]	59,463 (78.1%)	817 (1.35%)
	+ [d]	60,536 (79.6%)	1,041 (1.69%)
VarScan 2	–	46,646 (61.3%)	1,892 (3.90%)
	+ [c]	58,667 (77.1%)	775 (1.30%)
	+ [d]	59,403 (78.1%)	953 (1.58%)
GATK [b]	–	60,899 (80.0%)	2,622 (4.13%)
	+ [c]	58,699 (77.1%)	1,041 (1.74%)
	+ [d]	59,380 (78.0%)	1,228 (2.03%)
GATK [b] (+BQSR)	–	60,226 (79.1%)	2,421 (3.86%)
	+ [c]	58,277 (76.6%)	865 (1.46%)
	+ [d]	59,191 (77.8%)	1,016 (1.69%)
Atlas-Indel2	–	49,343 (64.8%)	1,946 (3.83%)
	+ [c]	59,124 (77.7%)	779 (1.30%)
	+ [d]	59,889 (78.7%)	934 (1.54%)

All conditions for filtering and calling homozygous indels are as in Table S13. Dindel failed to call indels for the alignment data pre-filtered using Coval-Refine with realignment, for unknown reasons.

[a] Variants called only with 'samtools pileup –vcf' command.

[b] Base quality score recalibration was applied using the artificially introduced SNP set.

[c] Coval-Refine without error correction (basic mode).

[d] Coval-Refine with error correction (error correction mode).
