# Supplementary material for: Coval: Improving Alignment Quality and Variant Calling Accuracy for Next-Generation Sequencing Data
Source: PLoS One. 2013 Oct 8;8(10):e75402. doi: 10.1371/journal.pone.0075402 (PMC3792961; doi:10.1371/journal.pone.0075402)
Supplement: Table S15 — Improvement by Coval-Refine of SNP/indel calling tools for mouse alignment data. (PDF) [file pone.0075402.s025.pdf]

Table S15. Improvement by Coval-Refine of SNP/indel calling tools for mouse alignment data.

Variant caller	Called variant	Coval-Refine	Calling accuracy	
			True positive rate	False positive rate
No caller	SNP	–	4,836,785 (96.5%)	1,877,035 (28.0%)
Coval-Call	SNP	–	4,654,552 (92.8%)	72,822 (1.54%)
		+ [c]	4,647,878 (92.7%)	16,839 (0.36%)
		+ [d]	4,653,518 (92.8%)	24,698 (0.53%)
SAMtools mpileup	SNP	–	4,430,660 (88.4%)	90,274 (2.00%)
		+ [c]	4,575,009 (91.2%)	23,618 (0.51%)
		+ [d]	4,552,660 (90.8%)	28,538 (0.62%)
Atlas-SNP2	SNP	–	4,561,307 (91.0%)	92,715 (1.99%)
		+ [c]	4,597,945 (91.7%)	40,697 (0.88%)
		+ [d]	4,578,811 (91.3%)	32,958 (0.71%)
VarScan [a]	SNP	–	4,734,803 (94.4%)	205,097 (4.15%)
		+ [c]	4,669,489 (93.1%)	35,389 (0.75%)
		+ [d]	4,689,635 (93.5%)	43,985 (0.93%)
GATK [b]	SNP	–	4,626,483 (92.3%)	112,109 (2.37%)
		+ [c]	4,589,868 (91.5%)	29,129 (0.63%)
		+ [d]	N.D.	N.D.
GeMS	SNP	–	4,640,525 (92.6%)	168,044 (3.49%)
		+ [c]	4,602,651 (91.8%)	41,354 (0.89%)
		+ [d]	4,622,410 (92.2%)	51,633 (1.10%)
No caller	indel	–	474,573 (94.6%)	2,598,724 (84.6%)
Coval-Call	indel	–	366,912 (73.1%)	28,546 (7.01%)
		+ [c]	447,105 (89.1%)	37,946 (7.80%)
		+ [d]	446,922 (89.0%)	49,654 (9.85%)
SAMtools mpileup	indel	–	461,097 (91.9%)	221,138 (29.8%)
		+ [c]	464,426 (92.5%)	149,438 (23.6%)
		+ [d]	469,097 (93.5%)	174,009 (25.7%)

VarScan	indel	–	389,490 (77.6%)	30,900 (7.35%)
		+[c]	422,716 (84.2%)	33,066 (7.25%)
		+[d]	424,791 (84.6%)	44,480 (9.48%)
GATK[a]	indel	–	461,497 (91.9%)	219,604 (32.2%)
		+[c]	453,294 (90.3%)	149,976 (24.9%)
		+[d]	N.D.	N.D.
AtlasIndel	indel	–	320,726 (63.9%)	36,363 (10.2%)
		+[c]	372,364 (74.2%)	21,992 (5.58%)
		+[d]	375,643 (74.8%)	23,591 (5.72%)

A simulated mouse genome was aligned with real mouse read data using BWA. The alignments were filtered (+) or not filtered (–) with Coval-Refine. Homozygous SNPs and indels were called with the indicated variant callers under the same conditions as in Tables S13 and S14.

[a] VarScan ver1 was used because of an unexpected error for VarScan ver2 with mouse data.

[b] A base quality score recalibration in the GATK v2.2 pipeline was omitted and a variant quality score recalibration replaced with simple filtering, as in Table S8. Analyses with Coval-Refine in the error correction mode were not conducted because of an unresolvable error.

[c] Coval-Refine in the basic mode.

[d] Coval-Refine in the error correction mode.
