# Supplementary material for: Coval: Improving Alignment Quality and Variant Calling Accuracy for Next-Generation Sequencing Data
Source: PLoS One. 2013 Oct 8;8(10):e75402. doi: 10.1371/journal.pone.0075402 (PMC3792961; doi:10.1371/journal.pone.0075402)
Supplement: Table S16 — SNP/indel calling accuracy of Coval for nematode alignment data. (PDF) [file pone.0075402.s026.pdf]

Table S16. SNP/indel calling accuracy of Coval for nematode alignment data.

Called variant	Coval-Refine	SNP/indel calling accuracy	
		True positive rate	False positive rate
SNP	–	187,635 (93.9%)	1,029 (0.55%)
	+ [a]	184,834 (92.5%)	541 (0.29%)
	+ [b]	184,679 (92.5%)	531 (0.29%)
Indel	–	15,506 (77.5%)	177 (1.13%)
	+ [a]	17,329 (86.7%)	229 (1.30%)
	+ [b]	17,299 (86.5%)	234 (1.33%)

A simulated nematode genome was aligned with nematode real (experimental) sequence data using BWA. The alignments were filtered (–) or not filtered (+) with Coval-Refine in the basic or error correction mode with 'maximum number of mismatch=3', and homozygous SNPs were called using Coval-Call with "minimum allele frequency=0.8" and "minimum number of reads supporting non-reference allele=2".

[a] Coval-Refine without error correction.

[b] Coval-Refine with error correction.
