# Supplementary material for: Coval: Improving Alignment Quality and Variant Calling Accuracy for Next-Generation Sequencing Data
Source: PLoS One. 2013 Oct 8;8(10):e75402. doi: 10.1371/journal.pone.0075402 (PMC3792961; doi:10.1371/journal.pone.0075402)
Supplement: Table S17 — Effect of filtering with alignment mapping quality for SNP/indel calling from targeted alignment data. (PDF) [file pone.0075402.s027.pdf]

Table S17. Effect of filtering with alignment mapping quality for SNP/indel calling from targeted alignment data.

Chromosome aligned [a]	Coval-Refine	Min-MQ [b]	SNP calling		Indel calling	
			TPR	FPR	TPR	FPR
All (383 Mb)	–	0	89.0	0.57	64.6	3.72
		1	87.2	0.69	64.2	3.75
		10	86.6	0.64	63.9	3.75
		20	85.2	0.58	61.7	3.60
	+	0	86.6	0.18	78.6	1.28
		1	84.8	0.21	77.9	1.28
Chr10 (23.7 Mb)	–	0	87.5	8.95	63.0	7.20
		1	86.9	9.56	63.6	8.56
		10	86.6	9.11	63.7	8.32
		20	86.8	5.96	63.0	7.64
		40	81.9	1.69	55.6	1.96
		60	80.4	1.45	55.8	2.40
	+ [c]	0	86.1	0.79	79.8	2.56
		20	83.9	0.63	76.8	2.24
Chr10-1M (1 Mb)	–	0	86.1	34.7	67.9	19.4
		1	86.3	35.6	67.9	19.4
		10	86.3	35.5	67.9	19.4
		20	87.2	30.4	67.3	17.8
		40	89.0	15.7	63.0	8.97
		60	89.1	14.3	63.6	7.89
	+ [c]	0	87.1	2.09	87.3	11.1
		60	85.4	1.43	79.4	4.38

[a] Chromosome regions of the simulated rice genome aligned with 75 bp paired-end reads sequenced from the whole rice genome. All, whole chromosomes; Chr10, chromosome 10; Chr10-1M, positions 1,000,001 to 2,000,000 of Chr10.

[b] Minimum mapping quality: reads with < min-MQ were filtered.

[c] Removal of the second paired-end mate read when the first mate is filtered and removal of a read pair that contained more than two total mismatches, with Coval-Refine in the basic mode.
